# Supplementary material for: Informed Bayesian survival analysis
Source: BMC Med Res Methodol. 2022 Sep 10;22:238. doi: 10.1186/s12874-022-01676-9 (PMC9464410; doi:10.1186/s12874-022-01676-9)
Supplement: Supplementary file 1 — Additional file 1. [file 12874_2022_1676_MOESM1_ESM.pdf]

Appendix:
Informed Bayesian survival analysis

April 9, 2022

Appendix A - Meta-Analytic Predictive Prior Distributions

This appendix describes how to obtain meta-analytic predictive prior distributions.

We used the historical participant level data ($k = 3$) to fit maximum likelihood versions of the survival models using the `flexsurv` `R` package [1]. We estimated the intercepts $\hat{\alpha}_{d,k}$ and auxiliary parameters $\hat{\gamma}_{d,k}$ and their standard errors $\text{se}(\hat{\alpha}_{d,k})$, $\text{se}(\hat{\gamma}_{d,k})$ for each data set and parametric family and combined them with a Bayesian random-effects meta-analytic model. We used wide Cauchy distributions on the meta-analytic pooled estimates ($\hat{\alpha}_d \sim \text{Cauchy}(0, 100)$ and $\hat{\gamma}_d \sim \text{Cauchy}(0, 100)$) and wide positive only Cauchy prior distributions for the heterogeneity estimates ($\tau_{\alpha,d} \sim \text{Cauchy}_+(0, 10)$ and $\tau_{\alpha,d} \sim \text{Cauchy}_+(0, 10)$) in order to warrant a convergence of the meta-analytic models with only three studies [2, 3, 4] while not introducing additional information. We used the `metaBMA` `R` package [5], to estimate the corresponding meta-analytic models,

$$\begin{aligned}\hat{\alpha}_{d,k} &\sim \text{Normal}(\hat{\alpha}_d,\ \text{se}(\hat{\alpha}_{d,k})^2 + \tau^2_{\alpha,d}),\\ \hat{\gamma}_{d,k} &\sim \text{Normal}(\hat{\gamma}_d,\ \text{se}(\hat{\gamma}_{d,k})^2 + \tau^2_{\gamma,d}),\end{aligned} \tag{1}$$

where $\hat{\alpha}_d$ and $\hat{\gamma}_d$ and correspond to the meta-analytic pooled estimates and $\tau_{\alpha,d}$ and $\tau_{\gamma,d}$ to the meta-analytic heterogeneity estimates. Subsequently, the meta-analytic predictive prior distributions for the intercepts and auxiliary parameters are,

$$\begin{aligned}\alpha_d &\sim \text{Normal}(\hat{\alpha_d},\ \text{se}(\hat{\alpha_d})^2 + \tau^2_{\alpha,d}),\\ \gamma_d &\sim \text{Normal}(\hat{\gamma_d},\ \text{se}(\hat{\gamma_d})^2 + \tau^2_{\gamma,d}),\end{aligned} \tag{2}$$

where $\text{se}(\hat{\alpha_d})$ corresponds to the standard error of the meta-analytic pooled estimate. Since the γ_d parameters are bounded to interval $(0, \infty)$, we proceed by estimating the meta-analytic model on a log scale which results in a log-normal meta-analytic prior predictive distributions.

Appendix B - Additional Simulation Results

This appendix contains additional results of the simulation study. Figure 1 visualizes the bias of log(AF) and bias of predicted survival at twenty years, Figure 2 visualizes the confidence interval coverage of log(AF), and Table 1 summarizes the probability and time of reaching each of the decisions.

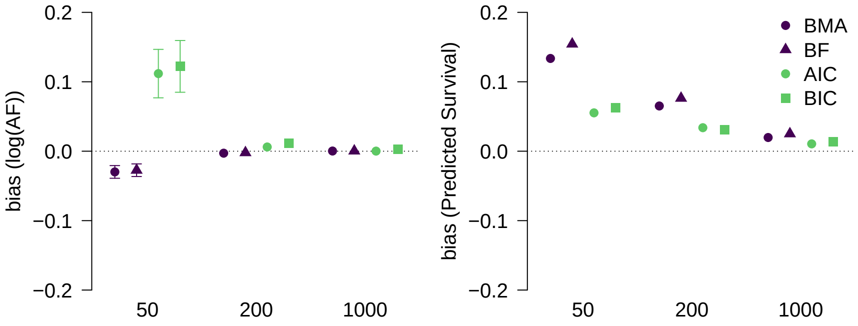

Figure 1: Left: Bias and 95% confidence intervals (y-axis; we do not show 95% confidence intervals in the case they are shorter than the symbols) of the mean acceleration factor estimates for different sample sizes (x-axis) and methods (colors/shapes) averaged across all simulation conditions. Right: Bias and 95% confidence intervals (y-axis) of the predicted mean survival at 20 years estimates for different sample sizes (x-axis) and methods (colors/shapes) averaged across all simulation conditions. Methods: Bayesian model-averaging (BMA, deep purple circles) and model selection over parametric families with: Bayes factors (BF = light green triangles), AIC (light green circles), and BIC (deep purple squares).

Table 1: Probability (SE) of finding support for the null hypothesis (H_0), alternative hypothesis (H_1), or not reaching a decision (undecided) in the simulated sequential analysis and the mean time in months (SE) of finding support for either the null or alternative hypothesis for each simulation condition comparing Bayesian model-averaging (BMA) to model selection using information (AIC, BIC), model selection using Bayes factors (BF), excluding the parametric models corresponding to the true data generating process. Results the model selection with AIC / BIC correspond to k = 20 steps in a sequential analysis with binding asymmetric boundaries, Hwang-Shih-DeCani spending function, and $\alpha = 0.05$ for one-sided test.

**Decision**	log(AF) = -.20			log(AF) = 0		
	H_0	Undecided	H_1	H_0	Undecided	H_1
BMA	0.998 (0.002)	0.000 (0.000)	0.002 (0.002)	0.896 (0.014)	0.048 (0.010)	0.056 (0.010)
BF	0.998 (0.002)	0.000 (0.000)	0.002 (0.002)	0.884 (0.014)	0.056 (0.010)	0.060 (0.011)
AIC	1.000 (0.000)	0.000 (0.000)	0.000 (0.000)	0.930 (0.011)	0.042 (0.009)	0.028 (0.007)
BIC	1.000 (0.000)	0.000 (0.000)	0.000 (0.000)	0.930 (0.011)	0.042 (0.009)	0.028 (0.007)

**Decision**	log(AF) = 0.20			log(AF) = 0.40		
	H_0	Undecided	H_1	H_0	Undecided	H_1
BMA	0.184 (0.017)	0.136 (0.015)	0.680 (0.021)	0.016 (0.006)	0.008 (0.004)	0.976 (0.007)
BF	0.164 (0.017)	0.152 (0.016)	0.684 (0.021)	0.016 (0.006)	0.008 (0.004)	0.976 (0.007)
AIC	0.112 (0.014)	0.212 (0.018)	0.676 (0.021)	0.006 (0.003)	0.012 (0.005)	0.982 (0.006)
BIC	0.116 (0.014)	0.208 (0.018)	0.676 (0.021)	0.006 (0.003)	0.012 (0.005)	0.982 (0.006)

**Time**	log(AF) = -0.20		log(AF) = 0		Average		
	H_0	H_1	H_0	H_0	H_0	H_1	H_0 or H_1
BMA	5.3 (0.14)	4.0.0 (–)	10.6 (0.36)	7.0 (0.96)	7.8 (0.21)	6.9 (0.93)	7.8 (0.20)
BF	5.6 (0.15)	4.0.0 (–)	11.4 (0.41)	8.1 (0.97)	8.3 (0.23)	7.9 (0.95)	8.3 (0.22)
AIC	8.5 (0.18)	0.0 (–)	19.6 (0.52)	29.3 (4.60)	13.9 (0.32)	29.3 (4.60)	14.1 (0.33)
BIC	8.4 (0.18)	0.0 (–)	19.6 (0.52)	29.0 (4.57)	13.8 (0.32)	29.0 (4.57)	14.0 (0.33)

**Time**	log(AF) = 0.20		log(AF) = 0.40		Average		
	H_0	H_1	H_0	H_0	H_0	H_1	H_0 or H_1
BMA	11.1 (0.76)	14.0 (0.55)	7.8 (1.31)	8.7 (0.27)	10.8 (0.71)	10.8 (0.29)	10.8 (0.27)
BF	11.6 (0.89)	13.9 (0.54)	8.8 (1.31)	8.7 (0.26)	11.4 (0.82)	10.8 (0.28)	10.9 (0.27)
AIC	29.7 (2.15)	28.5 (0.71)	12.5 (5.07)	16.9 (0.32)	28.8 (2.11)	21.6 (0.40)	22.1 (0.40)
BIC	28.9 (2.08)	28.3 (0.72)	11.9 (5.57)	16.6 (0.33)	28.1 (2.05)	21.4 (0.40)	21.9 (0.41)

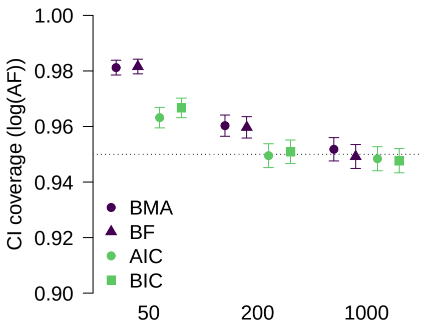

Figure 2: Confidence interval coverage and 95% confidence intervals (y-axis) of the mean acceleration factor estimates for different sample sizes (x-axis) and methods (colors/shapes) averaged across all simulation conditions. Methods: Bayesian model-averaging (BMA, deep purple circles) and model selection over parametric families with: Bayes factors (BF = light green triangles), AIC (light green circles), and BIC (deep purple squares).

References

[1] Jackson C. flexsurv: A platform for parametric survival modeling in R. Journal of Statistical Software. 2016;70(8):1–33.

[2] Williams DR, Rast P, Bürkner PC. Bayesian meta-analysis with weakly informative prior distributions; 2018. Available from: https://doi.org/10.31234/osf.io/7tbrm.

[3] Higgins JP, Thompson SG, Spiegelhalter DJ. A re-evaluation of random-effects meta-analysis. Journal of the Royal Statistical Society: Series A (Statistics in Society). 2009;172(1):137–159.

[4] Chung Y, Rabe-Hesketh S, Choi IH. Avoiding zero between-study variance estimates in random-effects meta-analysis. Statistics in Medicine. 2013;32(23):4071–4089. Available from: http://dx.doi.org/10.1002/sim.5821.

[5] Heck D, Gronau Q, Wagenmakers E. metaBMA: Bayesian model averaging for random-and fixed-effects meta-analysis [R Package]; 2017.
