# Supplementary material for: Informed Bayesian survival analysis
Source: BMC Med Res Methodol. 2022 Sep 10;22:238. doi: 10.1186/s12874-022-01676-9 (PMC9464410; doi:10.1186/s12874-022-01676-9)
Supplement: Supplementary file 2 — Additional file 2. [file 12874_2022_1676_MOESM2_ESM.pdf]

Supplementary Materials:
Informed Bayesian survival analysis

April 9, 2022

This document contains detailed simulation results of both the fixed-n and sequential design, and all performance measures, evaluated either when excluding models with the true parametric family corresponding to the data generating mechanism (Section 1) or when including all parametric families (Section 2).

1 Detailed Simulation Results Excluding the True Parametric Family

List of the included tables:

- Table 1 and 2: Probability of correctly recovering the true data generating parametric family across the simulation settings.
- Table 3 and 4: Bias of the log(AF) estimate (fixed-n).
- Table 5 and 6: RMSE of the log(AF) estimate (fixed-n).
- Table 7 and 8: Error rate and power when making decisions about the presence of the treatment effect (fixed-n).
- Table 9 and 10: Confidence interval coverage of the log(AF) estimate (fixed-n).
- Table 11 and 12: Bias of predicted survival at 20 years effect across the simulation settings (fixed-n).
- Table 13 and 14: RMSE of predicted survival at 20 years effect across the simulation settings (fixed-n).
- Table 15: Error rate and power when making decisions about the presence of the treatment effect (sequential-n).
- Table 16: Time to decide for the sequential design across the simulation settings.

Table 1: Probability of recovering the true parametric family (SE) for each simulation condition (Part 1) comparing Bayes factors (BF) to model selection using information (AIC, BIC). Rows correspond to different simulation conditions according to the true parametric family (Exp = exponential, Weib = Weibull, Log-N = log-normal, Log-L = Log-logistic, Gamma = gamma). Average corresponds to the results aggregated across distributions (last row), log(AF) $\times$ N (last column), or the whole simulation (bottom right corner).

**Bias**		log(AF) = -0.20			log(AF) = 0		
		N = 50	N = 200	N = 1000	N = 50	N = 200	N = 1000
Exp	BF	0.418 (0.022)	0.702 (0.020)	0.898 (0.014)	0.360 (0.021)	0.684 (0.021)	0.872 (0.015)
	AIC	0.690 (0.021)	0.730 (0.020)	0.750 (0.019)	0.690 (0.021)	0.732 (0.020)	0.742 (0.020)
	BIC	0.918 (0.012)	0.954 (0.009)	0.988 (0.005)	0.894 (0.014)	0.966 (0.008)	0.984 (0.006)
Weib	BF	0.018 (0.006)	0.114 (0.014)	0.528 (0.022)	0.030 (0.008)	0.078 (0.012)	0.454 (0.022)
	AIC	0.104 (0.014)	0.234 (0.019)	0.558 (0.022)	0.116 (0.014)	0.222 (0.019)	0.460 (0.022)
	BIC	0.050 (0.010)	0.084 (0.012)	0.356 (0.021)	0.048 (0.010)	0.062 (0.011)	0.270 (0.020)
Log-N	BF	0.652 (0.021)	0.762 (0.019)	0.942 (0.010)	0.672 (0.021)	0.784 (0.018)	0.920 (0.012)
	AIC	0.006 (0.003)	0.050 (0.010)	0.012 (0.005)	0.006 (0.003)	0.040 (0.009)	0.016 (0.006)
	BIC	0.002 (0.002)	0.018 (0.006)	0.012 (0.005)	0.000 (0.000)	0.014 (0.005)	0.014 (0.005)
Log-L	BF	0.010 (0.004)	0.072 (0.012)	0.458 (0.022)	0.004 (0.003)	0.066 (0.011)	0.480 (0.022)
	AIC	0.288 (0.020)	0.342 (0.021)	0.118 (0.014)	0.270 (0.020)	0.336 (0.021)	0.134 (0.015)
	BIC	0.118 (0.014)	0.152 (0.016)	0.116 (0.014)	0.116 (0.014)	0.142 (0.016)	0.120 (0.015)
Gamma	BF	0.060 (0.011)	0.088 (0.013)	0.146 (0.016)	0.076 (0.012)	0.086 (0.013)	0.124 (0.015)
	AIC	0.044 (0.009)	0.136 (0.015)	0.270 (0.020)	0.034 (0.008)	0.088 (0.013)	0.270 (0.020)
	BIC	0.010 (0.004)	0.058 (0.010)	0.222 (0.019)	0.008 (0.004)	0.038 (0.009)	0.208 (0.018)
Average	BF	0.232 (0.008)	0.348 (0.010)	0.594 (0.010)	0.228 (0.008)	0.340 (0.009)	0.570 (0.010)
	AIC	0.226 (0.008)	0.298 (0.009)	0.342 (0.009)	0.223 (0.008)	0.284 (0.009)	0.324 (0.009)
	BIC	0.220 (0.008)	0.253 (0.009)	0.339 (0.009)	0.213 (0.008)	0.244 (0.009)	0.319 (0.009)

Table 2: Probability of recovering the true parametric family (SE) for each simulation condition (Part 2) comparing Bayes factors (BF) to model selection using information (AIC, BIC). Rows correspond to different simulation conditions according to the true parametric family (Exp = exponential, Weib = Weibull, Log-N = log-normal, Log-L = Log-logistic, Gamma = gamma). Average corresponds to the results aggregated across distributions (last row), log(AF) $\times$ N (last column), or the whole simulation (bottom right corner).

**Bias**		log(AF) = 0.20			log(AF) = 0.40			Average
		N = 50	N = 200	N = 1000	N = 50	N = 200	N = 1000	
Exp	BF	0.376 (0.022)	0.670 (0.021)	0.860 (0.016)	0.348 (0.021)	0.644 (0.021)	0.856 (0.016)	0.641 (0.006)
	AIC	0.710 (0.020)	0.690 (0.021)	0.740 (0.020)	0.696 (0.021)	0.706 (0.020)	0.726 (0.020)	0.717 (0.006)
	BIC	0.914 (0.013)	0.960 (0.009)	0.972 (0.007)	0.934 (0.011)	0.964 (0.008)	0.974 (0.007)	0.952 (0.003)
Weib	BF	0.020 (0.006)	0.058 (0.010)	0.444 (0.022)	0.036 (0.008)	0.046 (0.009)	0.472 (0.022)	0.192 (0.005)
	AIC	0.092 (0.013)	0.182 (0.017)	0.478 (0.022)	0.064 (0.011)	0.162 (0.016)	0.488 (0.022)	0.263 (0.006)
	BIC	0.048 (0.010)	0.052 (0.010)	0.266 (0.020)	0.026 (0.007)	0.054 (0.010)	0.256 (0.020)	0.131 (0.004)
Log-N	BF	0.682 (0.021)	0.800 (0.018)	0.928 (0.012)	0.692 (0.021)	0.770 (0.019)	0.894 (0.014)	0.791 (0.005)
	AIC	0.002 (0.002)	0.052 (0.010)	0.018 (0.006)	0.004 (0.003)	0.034 (0.008)	0.022 (0.007)	0.022 (0.002)
	BIC	0.002 (0.002)	0.008 (0.004)	0.018 (0.006)	0.004 (0.003)	0.016 (0.006)	0.022 (0.007)	0.011 (0.001)
Log-L	BF	0.004 (0.003)	0.064 (0.011)	0.398 (0.022)	0.002 (0.002)	0.064 (0.011)	0.388 (0.022)	0.168 (0.005)
	AIC	0.258 (0.020)	0.374 (0.022)	0.158 (0.016)	0.260 (0.020)	0.290 (0.020)	0.168 (0.017)	0.250 (0.006)
	BIC	0.098 (0.013)	0.146 (0.016)	0.148 (0.016)	0.100 (0.013)	0.140 (0.016)	0.146 (0.016)	0.128 (0.004)
Gamma	BF	0.062 (0.011)	0.074 (0.012)	0.130 (0.015)	0.048 (0.010)	0.076 (0.012)	0.112 (0.014)	0.090 (0.004)
	AIC	0.018 (0.006)	0.122 (0.015)	0.298 (0.020)	0.022 (0.007)	0.110 (0.014)	0.318 (0.021)	0.144 (0.005)
	BIC	0.010 (0.004)	0.058 (0.010)	0.238 (0.019)	0.006 (0.003)	0.026 (0.007)	0.218 (0.018)	0.092 (0.004)
Average	BF	0.229 (0.008)	0.333 (0.009)	0.552 (0.010)	0.225 (0.008)	0.320 (0.009)	0.544 (0.010)	0.376 (0.003)
	AIC	0.216 (0.008)	0.284 (0.009)	0.338 (0.009)	0.209 (0.008)	0.260 (0.009)	0.344 (0.010)	0.279 (0.003)
	BIC	0.214 (0.008)	0.245 (0.009)	0.328 (0.009)	0.214 (0.008)	0.240 (0.009)	0.323 (0.009)	0.263 (0.003)

Table 3: Bias (SE) for each simulation condition (Part 1) comparing Bayesian model-averaging (BMA) to model selection using information (AIC, BIC), model selection using Bayes factors (BF), and Bayesian or frequentist survival model with the true data generating survival function (Oracle), excluding the parametric models corresponding to the true data generating process. Rows correspond to different simulation conditions according to the true parametric family (Exp = exponential, Weib = Weibull, Log-N = log-normal, Log-L = Log-logistic, Gamma = gamma). Average corresponds to the results aggregated across distributions (last row), log(AF) $\times$ N (last column), or the whole simulation (bottom right corner).

**Bias**		log(AF) = -0.20			log(AF) = 0		
		N = 50	N = 200	N = 1000	N = 50	N = 200	N = 1000
Exp	BMA	0.041 (0.021)	-0.034 (0.012)	0.006 (0.006)	0.018 (0.021)	0.019 (0.014)	-0.004 (0.007)
	AIC	-0.009 (0.035)	-0.044 (0.013)	0.007 (0.006)	0.033 (0.054)	0.023 (0.015)	-0.003 (0.007)
	BIC	-0.009 (0.035)	-0.044 (0.013)	0.007 (0.006)	0.033 (0.054)	0.023 (0.015)	-0.003 (0.007)
	BF	0.044 (0.021)	-0.030 (0.012)	0.007 (0.006)	0.024 (0.021)	0.021 (0.014)	-0.004 (0.007)
	Oracle (Bayes)	0.036 (0.021)	-0.022 (0.011)	0.012 (0.006)	0.013 (0.021)	0.017 (0.013)	-0.003 (0.006)
	Oracle (freq)	-0.008 (0.066)	-0.032 (0.012)	0.010 (0.006)	-0.006 (0.077)	0.024 (0.014)	-0.002 (0.006)
Weib	BMA	-0.022 (0.019)	0.002 (0.012)	-0.008 (0.005)	-0.005 (0.021)	0.000 (0.012)	-0.004 (0.006)
	AIC	-0.076 (0.025)	0.004 (0.012)	-0.003 (0.005)	-0.005 (0.028)	0.005 (0.013)	-0.002 (0.005)
	BIC	-0.087 (0.026)	-0.005 (0.013)	-0.009 (0.005)	0.001 (0.028)	0.005 (0.013)	-0.002 (0.006)
	BF	-0.018 (0.019)	0.005 (0.012)	-0.006 (0.005)	-0.003 (0.020)	0.001 (0.012)	-0.003 (0.006)
	Oracle (Bayes)	-0.020 (0.019)	0.008 (0.012)	-0.001 (0.005)	-0.006 (0.021)	0.000 (0.012)	-0.003 (0.005)
	Oracle (freq)	-0.050 (0.023)	0.017 (0.011)	0.003 (0.005)	-0.006 (0.026)	0.005 (0.012)	-0.002 (0.005)
Log-N	BMA	0.020 (0.021)	0.010 (0.012)	0.021 (0.005)	0.004 (0.020)	0.018 (0.013)	0.000 (0.005)
	AIC	-0.021 (0.088)	0.010 (0.012)	0.021 (0.005)	0.102 (0.064)	0.022 (0.013)	0.001 (0.005)
	BIC	-0.036 (0.088)	0.001 (0.013)	0.020 (0.005)	0.094 (0.065)	0.024 (0.014)	0.001 (0.005)
	BF	0.019 (0.021)	0.010 (0.012)	0.019 (0.005)	0.002 (0.020)	0.019 (0.013)	0.000 (0.005)
	Oracle (Bayes)	0.013 (0.020)	-0.003 (0.012)	0.007 (0.005)	0.001 (0.020)	0.017 (0.013)	0.000 (0.006)
	Oracle (freq)	-0.018 (0.042)	-0.002 (0.012)	0.008 (0.005)	0.059 (0.039)	0.024 (0.013)	0.002 (0.006)
Log-L	BMA	-0.010 (0.021)	-0.015 (0.012)	-0.009 (0.005)	-0.009 (0.021)	0.013 (0.012)	0.002 (0.005)
	AIC	-0.074 (0.039)	-0.013 (0.013)	-0.005 (0.005)	0.040 (0.051)	0.020 (0.013)	0.003 (0.005)
	BIC	-0.104 (0.051)	-0.020 (0.013)	-0.009 (0.005)	0.009 (0.066)	0.020 (0.013)	0.003 (0.005)
	BF	-0.007 (0.020)	-0.012 (0.012)	-0.008 (0.005)	-0.006 (0.021)	0.015 (0.012)	0.003 (0.005)
	Oracle (Bayes)	-0.005 (0.021)	-0.011 (0.012)	-0.013 (0.005)	-0.006 (0.021)	0.014 (0.012)	0.003 (0.005)
	Oracle (freq)	-0.061 (0.031)	-0.007 (0.012)	-0.011 (0.005)	0.013 (0.034)	0.018 (0.012)	0.004 (0.005)
Gamma	BMA	0.011 (0.019)	-0.005 (0.012)	-0.003 (0.005)	0.006 (0.020)	-0.014 (0.012)	0.006 (0.005)
	AIC	-0.018 (0.026)	-0.001 (0.012)	0.002 (0.005)	0.009 (0.029)	-0.009 (0.012)	0.007 (0.005)
	BIC	-0.035 (0.026)	-0.012 (0.013)	-0.002 (0.005)	0.017 (0.030)	-0.009 (0.013)	0.008 (0.006)
	BF	0.019 (0.019)	0.002 (0.012)	-0.001 (0.005)	0.006 (0.020)	-0.013 (0.012)	0.007 (0.005)
	Oracle (Bayes)	0.011 (0.019)	0.001 (0.011)	0.000 (0.005)	0.010 (0.020)	-0.015 (0.012)	0.006 (0.005)
	Oracle (freq)	-0.004 (0.024)	0.009 (0.011)	0.003 (0.005)	0.017 (0.026)	-0.008 (0.012)	0.007 (0.005)
Average	BMA	0.008 (0.009)	-0.008 (0.005)	0.001 (0.002)	0.003 (0.009)	0.007 (0.006)	0.000 (0.003)
	AIC	-0.040 (0.022)	-0.009 (0.006)	0.004 (0.002)	0.036 (0.021)	0.012 (0.006)	0.001 (0.002)
	BIC	-0.054 (0.023)	-0.016 (0.006)	0.002 (0.002)	0.031 (0.023)	0.013 (0.006)	0.001 (0.003)
	BF	0.012 (0.009)	-0.005 (0.005)	0.002 (0.002)	0.005 (0.009)	0.009 (0.006)	0.000 (0.003)
	Oracle (Bayes)	0.007 (0.009)	-0.005 (0.005)	0.001 (0.002)	0.003 (0.009)	0.007 (0.005)	0.000 (0.002)
	Oracle (freq)	-0.028 (0.018)	-0.003 (0.005)	0.003 (0.002)	0.015 (0.020)	0.013 (0.006)	0.002 (0.002)

Table 4: Bias (SE) for each simulation condition (Part 2) comparing Bayesian model-averaging (BMA) to model selection using information (AIC, BIC), model selection using Bayes factors (BF), and Bayesian or frequentist survival model with the true data generating survival function (Oracle), excluding the parametric models corresponding to the true data generating process. Rows correspond to different simulation conditions according to the true parametric family (Exp = exponential, Weib = Weibull, Log-N = log-normal, Log-L = Log-logistic, Gamma = gamma). Average corresponds to the results aggregated across distributions (last row), log(AF) × N (last column), or the whole simulation (bottom right corner).

**Bias**		log(AF) = 0.20			log(AF) = 0.40			Average
		N = 50	N = 200	N = 1000	N = 50	N = 200	N = 1000	
Exp	BMA	-0.067 (0.023)	-0.005 (0.014)	-0.009 (0.007)	-0.097 (0.022)	-0.036 (0.015)	-0.009 (0.007)	-0.015 (0.004)
	AIC	0.110 (0.089)	0.017 (0.016)	-0.006 (0.007)	0.234 (0.083)	-0.002 (0.017)	-0.005 (0.007)	0.030 (0.012)
	BIC	0.110 (0.089)	0.017 (0.016)	-0.006 (0.007)	0.234 (0.083)	-0.002 (0.017)	-0.005 (0.007)	0.030 (0.012)
	BF	-0.063 (0.022)	-0.003 (0.014)	-0.009 (0.007)	-0.092 (0.022)	-0.037 (0.015)	-0.010 (0.007)	-0.013 (0.004)
	Oracle (Bayes)	-0.072 (0.023)	-0.016 (0.013)	-0.015 (0.006)	-0.105 (0.022)	-0.052 (0.014)	-0.021 (0.007)	-0.019 (0.004)
	Oracle (freq)	0.222 (0.120)	0.005 (0.015)	-0.011 (0.006)	0.313 (0.138)	-0.013 (0.016)	-0.014 (0.007)	0.041 (0.018)
Weib	BMA	-0.060 (0.019)	0.007 (0.012)	0.010 (0.006)	-0.100 (0.022)	0.027 (0.013)	0.019 (0.006)	-0.011 (0.004)
	AIC	0.100 (0.076)	0.020 (0.013)	0.007 (0.006)	0.256 (0.105)	0.048 (0.014)	0.011 (0.006)	0.030 (0.011)
	BIC	0.101 (0.076)	0.028 (0.013)	0.015 (0.006)	0.338 (0.119)	0.073 (0.015)	0.031 (0.006)	0.041 (0.013)
	BF	-0.055 (0.019)	0.006 (0.012)	0.008 (0.006)	-0.096 (0.021)	0.023 (0.013)	0.017 (0.006)	-0.010 (0.004)
	Oracle (Bayes)	-0.060 (0.020)	0.003 (0.012)	0.001 (0.006)	-0.099 (0.022)	0.021 (0.013)	0.004 (0.005)	-0.013 (0.004)
	Oracle (freq)	0.062 (0.063)	0.004 (0.012)	-0.001 (0.006)	0.224 (0.102)	0.020 (0.013)	-0.001 (0.005)	0.023 (0.011)
Log-N	BMA	-0.038 (0.021)	-0.010 (0.012)	-0.020 (0.005)	-0.105 (0.021)	-0.047 (0.013)	-0.029 (0.006)	-0.015 (0.004)
	AIC	-0.001 (0.089)	-0.002 (0.012)	-0.018 (0.005)	0.203 (0.109)	-0.038 (0.013)	-0.027 (0.006)	0.021 (0.015)
	BIC	-0.015 (0.098)	0.013 (0.013)	-0.017 (0.005)	0.328 (0.129)	-0.008 (0.014)	-0.026 (0.006)	0.032 (0.016)
	BF	-0.037 (0.021)	-0.009 (0.012)	-0.017 (0.005)	-0.106 (0.021)	-0.048 (0.013)	-0.025 (0.006)	-0.014 (0.004)
	Oracle (Bayes)	-0.045 (0.021)	-0.002 (0.012)	-0.003 (0.005)	-0.105 (0.021)	-0.029 (0.013)	0.002 (0.006)	-0.012 (0.004)
	Oracle (freq)	0.001 (0.041)	0.010 (0.012)	-0.002 (0.005)	0.180 (0.101)	-0.011 (0.014)	0.003 (0.006)	0.021 (0.011)
Log-L	BMA	-0.036 (0.020)	-0.013 (0.012)	0.008 (0.006)	-0.064 (0.021)	-0.002 (0.013)	-0.009 (0.006)	-0.012 (0.004)
	AIC	0.143 (0.076)	0.001 (0.013)	0.006 (0.006)	0.485 (0.130)	0.010 (0.014)	-0.014 (0.006)	0.050 (0.014)
	BIC	0.147 (0.076)	0.002 (0.013)	0.012 (0.006)	0.583 (0.143)	0.034 (0.014)	-0.003 (0.006)	0.056 (0.016)
	BF	-0.030 (0.020)	-0.011 (0.012)	0.007 (0.006)	-0.068 (0.021)	-0.006 (0.013)	-0.009 (0.006)	-0.011 (0.004)
	Oracle (Bayes)	-0.033 (0.020)	-0.014 (0.012)	0.008 (0.006)	-0.064 (0.021)	-0.009 (0.013)	-0.010 (0.006)	-0.012 (0.004)
	Oracle (freq)	0.139 (0.099)	-0.008 (0.012)	0.008 (0.005)	0.251 (0.072)	-0.005 (0.013)	-0.012 (0.006)	0.027 (0.011)
Gamma	BMA	-0.020 (0.021)	0.006 (0.013)	0.017 (0.006)	-0.064 (0.021)	0.023 (0.013)	0.020 (0.006)	-0.001 (0.004)
	AIC	0.261 (0.106)	0.013 (0.013)	0.012 (0.006)	0.463 (0.132)	0.037 (0.014)	0.011 (0.006)	0.066 (0.015)
	BIC	0.253 (0.112)	0.025 (0.014)	0.018 (0.006)	0.480 (0.133)	0.065 (0.014)	0.025 (0.006)	0.070 (0.015)
	BF	-0.015 (0.021)	0.005 (0.012)	0.015 (0.006)	-0.067 (0.021)	0.019 (0.013)	0.017 (0.006)	-0.001 (0.004)
	Oracle (Bayes)	-0.021 (0.021)	-0.004 (0.012)	0.010 (0.005)	-0.063 (0.021)	0.008 (0.012)	0.007 (0.006)	-0.004 (0.004)
	Oracle (freq)	0.102 (0.049)	0.001 (0.012)	0.010 (0.005)	0.169 (0.062)	0.012 (0.013)	0.005 (0.006)	0.027 (0.008)
Average	BMA	-0.044 (0.009)	-0.003 (0.006)	0.001 (0.003)	-0.086 (0.010)	-0.007 (0.006)	-0.001 (0.003)	-0.011 (0.002)
	AIC	0.123 (0.039)	0.010 (0.006)	0.000 (0.003)	0.328 (0.051)	0.011 (0.007)	-0.005 (0.003)	0.039 (0.006)
	BIC	0.119 (0.041)	0.017 (0.006)	0.004 (0.003)	0.393 (0.055)	0.032 (0.007)	0.004 (0.003)	0.046 (0.007)
	BF	-0.040 (0.009)	-0.002 (0.006)	0.001 (0.003)	-0.086 (0.010)	-0.010 (0.006)	-0.002 (0.003)	-0.010 (0.002)
	Oracle (Bayes)	-0.046 (0.009)	-0.006 (0.005)	0.000 (0.003)	-0.087 (0.010)	-0.012 (0.006)	-0.004 (0.003)	-0.012 (0.002)
	Oracle (freq)	0.105 (0.036)	0.002 (0.006)	0.001 (0.003)	0.227 (0.044)	0.001 (0.006)	-0.004 (0.003)	0.028 (0.006)

Table 5: RMSE (SE) for each simulation condition (Part 1) comparing Bayesian model-averaging (BMA) to model selection using information (AIC, BIC), model selection using Bayes factors (BF), and Bayesian or frequentist survival model with the true data generating survival function (Oracle), excluding the parametric models corresponding to the true data generating process. Rows correspond to different simulation conditions according to the true parametric family (Exp = exponential, Weib = Weibull, Log-N = log-normal, Log-L = Log-logistic, Gamma = gamma). Average corresponds to the results aggregated across distributions (last row), log(AF) $\times$ N (last column), or the whole simulation (bottom right corner).

**RMSE**		log(AF) = -0.20			log(AF) = 0		
		N = 50	N = 200	N = 1000	N = 50	N = 200	N = 1000
Exp	BMA	0.468 (0.013)	0.266 (0.009)	0.138 (0.004)	0.470 (0.014)	0.306 (0.009)	0.147 (0.004)
	AIC	0.790 (0.051)	0.291 (0.010)	0.139 (0.004)	1.211 (0.328)	0.333 (0.011)	0.148 (0.004)
	BIC	0.790 (0.051)	0.291 (0.010)	0.139 (0.004)	1.211 (0.328)	0.333 (0.011)	0.148 (0.004)
	BF	0.466 (0.013)	0.265 (0.009)	0.138 (0.004)	0.470 (0.014)	0.306 (0.009)	0.147 (0.004)
	Oracle (Bayes)	0.460 (0.013)	0.247 (0.008)	0.134 (0.004)	0.466 (0.014)	0.287 (0.009)	0.142 (0.004)
	Oracle (freq)	1.465 (0.471)	0.268 (0.009)	0.136 (0.004)	1.723 (0.454)	0.311 (0.010)	0.144 (0.004)
Weib	BMA	0.421 (0.013)	0.267 (0.010)	0.115 (0.004)	0.458 (0.014)	0.269 (0.009)	0.125 (0.004)
	AIC	0.558 (0.019)	0.277 (0.012)	0.113 (0.004)	0.631 (0.024)	0.280 (0.010)	0.123 (0.004)
	BIC	0.591 (0.023)	0.288 (0.011)	0.117 (0.004)	0.635 (0.023)	0.289 (0.010)	0.127 (0.004)
	BF	0.418 (0.013)	0.265 (0.010)	0.115 (0.004)	0.456 (0.014)	0.270 (0.009)	0.124 (0.004)
	Oracle (Bayes)	0.423 (0.013)	0.258 (0.010)	0.111 (0.004)	0.459 (0.014)	0.264 (0.009)	0.119 (0.004)
	Oracle (freq)	0.521 (0.023)	0.255 (0.010)	0.109 (0.003)	0.579 (0.023)	0.258 (0.009)	0.118 (0.004)
Log-N	BMA	0.468 (0.017)	0.257 (0.008)	0.113 (0.004)	0.456 (0.014)	0.281 (0.009)	0.116 (0.004)
	AIC	1.965 (0.474)	0.264 (0.008)	0.113 (0.004)	1.433 (0.473)	0.287 (0.010)	0.116 (0.004)
	BIC	1.977 (0.471)	0.280 (0.008)	0.114 (0.004)	1.447 (0.467)	0.305 (0.011)	0.117 (0.004)
	BF	0.469 (0.017)	0.258 (0.008)	0.113 (0.004)	0.458 (0.014)	0.281 (0.010)	0.117 (0.004)
	Oracle (Bayes)	0.453 (0.018)	0.262 (0.008)	0.116 (0.004)	0.447 (0.014)	0.284 (0.010)	0.123 (0.004)
	Oracle (freq)	0.929 (0.163)	0.272 (0.008)	0.117 (0.004)	0.870 (0.186)	0.295 (0.010)	0.123 (0.004)
Log-L	BMA	0.462 (0.016)	0.273 (0.010)	0.114 (0.004)	0.464 (0.015)	0.273 (0.009)	0.114 (0.004)
	AIC	0.872 (0.220)	0.282 (0.010)	0.113 (0.004)	1.147 (0.450)	0.285 (0.010)	0.114 (0.004)
	BIC	1.148 (0.476)	0.294 (0.011)	0.115 (0.004)	1.467 (0.457)	0.294 (0.010)	0.115 (0.004)
	BF	0.457 (0.016)	0.275 (0.010)	0.114 (0.004)	0.460 (0.015)	0.276 (0.009)	0.116 (0.004)
	Oracle (Bayes)	0.460 (0.016)	0.270 (0.010)	0.116 (0.004)	0.464 (0.015)	0.269 (0.009)	0.115 (0.004)
	Oracle (freq)	0.703 (0.091)	0.273 (0.010)	0.116 (0.004)	0.765 (0.109)	0.271 (0.009)	0.115 (0.004)
Gamma	BMA	0.428 (0.014)	0.263 (0.009)	0.111 (0.004)	0.457 (0.014)	0.270 (0.009)	0.122 (0.004)
	AIC	0.581 (0.026)	0.272 (0.009)	0.109 (0.004)	0.648 (0.027)	0.279 (0.010)	0.120 (0.004)
	BIC	0.591 (0.023)	0.280 (0.009)	0.112 (0.004)	0.669 (0.027)	0.288 (0.010)	0.123 (0.004)
	BF	0.425 (0.014)	0.262 (0.008)	0.110 (0.004)	0.455 (0.014)	0.270 (0.009)	0.121 (0.004)
	Oracle (Bayes)	0.426 (0.014)	0.251 (0.008)	0.109 (0.004)	0.454 (0.014)	0.259 (0.009)	0.118 (0.004)
	Oracle (freq)	0.547 (0.028)	0.251 (0.008)	0.108 (0.003)	0.591 (0.025)	0.259 (0.009)	0.118 (0.004)
Average	BMA	0.450 (0.007)	0.265 (0.004)	0.119 (0.002)	0.461 (0.006)	0.280 (0.004)	0.125 (0.002)
	AIC	1.086 (0.171)	0.277 (0.004)	0.118 (0.002)	1.063 (0.158)	0.293 (0.004)	0.125 (0.002)
	BIC	1.145 (0.177)	0.287 (0.005)	0.120 (0.002)	1.146 (0.165)	0.302 (0.005)	0.127 (0.002)
	BF	0.447 (0.007)	0.265 (0.004)	0.118 (0.002)	0.460 (0.006)	0.281 (0.004)	0.126 (0.002)
	Oracle (Bayes)	0.445 (0.007)	0.258 (0.004)	0.118 (0.002)	0.458 (0.006)	0.273 (0.004)	0.124 (0.002)
	Oracle (freq)	0.903 (0.148)	0.264 (0.004)	0.118 (0.002)	1.000 (0.155)	0.280 (0.004)	0.124 (0.002)

Table 6: RMSE (SE) for each simulation condition (Part 2) comparing Bayesian model-averaging (BMA) to model selection using information (AIC, BIC), model selection using Bayes factors (BF), and Bayesian or frequentist survival model with the true data generating survival function (Oracle), excluding the parametric models corresponding to the true data generating process. Rows correspond to different simulation conditions according to the true parametric family (Exp = exponential, Weib = Weibull, Log-N = log-normal, Log-L = Log-logistic, Gamma = gamma). Average corresponds to the results aggregated across distributions (last row), log(AF) × N (last column), or the whole simulation (bottom right corner).

**RMSE**		log(AF) = 0.20			log(AF) = 0.40			Average
		N = 50	N = 200	N = 1000	N = 50	N = 200	N = 1000	
Exp	BMA	0.510 (0.016)	0.315 (0.011)	0.147 (0.005)	0.506 (0.015)	0.335 (0.012)	0.155 (0.005)	0.344 (0.004)
	AIC	1.980 (0.426)	0.353 (0.014)	0.149 (0.005)	1.878 (0.266)	0.382 (0.016)	0.157 (0.005)	0.917 (0.092)
	BIC	1.980 (0.426)	0.353 (0.014)	0.149 (0.005)	1.878 (0.266)	0.382 (0.016)	0.157 (0.005)	0.917 (0.092)
	BF	0.505 (0.017)	0.314 (0.011)	0.147 (0.005)	0.505 (0.016)	0.336 (0.012)	0.154 (0.005)	0.343 (0.004)
	Oracle (Bayes)	0.512 (0.017)	0.296 (0.011)	0.140 (0.004)	0.508 (0.015)	0.318 (0.011)	0.148 (0.005)	0.337 (0.004)
	Oracle (freq)	2.683 (0.448)	0.324 (0.012)	0.142 (0.004)	3.104 (0.445)	0.348 (0.012)	0.150 (0.005)	1.367 (0.125)
Weib	BMA	0.437 (0.014)	0.274 (0.009)	0.131 (0.004)	0.493 (0.015)	0.294 (0.010)	0.128 (0.005)	0.315 (0.004)
	AIC	1.697 (0.462)	0.294 (0.011)	0.129 (0.004)	2.351 (0.446)	0.323 (0.013)	0.125 (0.005)	0.891 (0.119)
	BIC	1.704 (0.459)	0.295 (0.010)	0.136 (0.004)	2.684 (0.451)	0.337 (0.012)	0.135 (0.005)	0.970 (0.122)
	BF	0.433 (0.014)	0.274 (0.009)	0.130 (0.004)	0.487 (0.014)	0.294 (0.010)	0.127 (0.005)	0.313 (0.004)
	Oracle (Bayes)	0.440 (0.014)	0.268 (0.009)	0.125 (0.004)	0.494 (0.014)	0.291 (0.010)	0.121 (0.004)	0.313 (0.004)
	Oracle (freq)	1.410 (0.377)	0.270 (0.011)	0.123 (0.004)	2.290 (0.441)	0.297 (0.011)	0.120 (0.004)	0.825 (0.112)
Log-N	BMA	0.472 (0.013)	0.265 (0.008)	0.114 (0.003)	0.485 (0.015)	0.290 (0.009)	0.133 (0.004)	0.322 (0.004)
	AIC	1.993 (0.451)	0.271 (0.009)	0.114 (0.003)	2.442 (0.431)	0.302 (0.010)	0.133 (0.004)	1.163 (0.124)
	BIC	2.183 (0.459)	0.296 (0.010)	0.115 (0.003)	2.896 (0.439)	0.321 (0.011)	0.134 (0.004)	1.277 (0.125)
	BF	0.473 (0.013)	0.266 (0.008)	0.115 (0.003)	0.488 (0.015)	0.289 (0.009)	0.133 (0.004)	0.322 (0.004)
	Oracle (Bayes)	0.460 (0.013)	0.267 (0.008)	0.119 (0.004)	0.474 (0.015)	0.294 (0.009)	0.135 (0.004)	0.318 (0.004)
	Oracle (freq)	0.906 (0.123)	0.277 (0.009)	0.119 (0.004)	2.268 (0.855)	0.308 (0.010)	0.135 (0.004)	0.816 (0.184)
Log-L	BMA	0.456 (0.016)	0.276 (0.008)	0.125 (0.004)	0.473 (0.015)	0.292 (0.009)	0.132 (0.004)	0.320 (0.004)
	AIC	1.693 (0.454)	0.290 (0.009)	0.125 (0.004)	2.955 (0.429)	0.309 (0.011)	0.133 (0.004)	1.083 (0.118)
	BIC	1.703 (0.451)	0.297 (0.009)	0.129 (0.004)	3.253 (0.437)	0.323 (0.011)	0.138 (0.004)	1.203 (0.122)
	BF	0.451 (0.016)	0.276 (0.008)	0.126 (0.004)	0.467 (0.015)	0.294 (0.010)	0.132 (0.004)	0.318 (0.004)
	Oracle (Bayes)	0.455 (0.016)	0.271 (0.008)	0.123 (0.004)	0.472 (0.015)	0.287 (0.009)	0.131 (0.004)	0.318 (0.004)
	Oracle (freq)	2.222 (1.366)	0.276 (0.009)	0.123 (0.004)	1.633 (0.246)	0.293 (0.010)	0.130 (0.004)	0.868 (0.236)
Gamma	BMA	0.472 (0.017)	0.280 (0.010)	0.128 (0.004)	0.480 (0.016)	0.290 (0.009)	0.129 (0.004)	0.318 (0.004)
	AIC	2.389 (0.440)	0.287 (0.010)	0.124 (0.004)	2.992 (0.444)	0.310 (0.011)	0.126 (0.004)	1.148 (0.122)
	BIC	2.516 (0.450)	0.303 (0.010)	0.129 (0.004)	2.998 (0.443)	0.327 (0.011)	0.136 (0.004)	1.174 (0.123)
	BF	0.472 (0.017)	0.278 (0.009)	0.127 (0.004)	0.476 (0.016)	0.287 (0.009)	0.128 (0.004)	0.316 (0.004)
	Oracle (Bayes)	0.470 (0.017)	0.269 (0.009)	0.123 (0.004)	0.477 (0.016)	0.279 (0.009)	0.124 (0.004)	0.313 (0.004)
	Oracle (freq)	1.104 (0.165)	0.267 (0.009)	0.122 (0.004)	1.396 (0.196)	0.283 (0.009)	0.123 (0.004)	0.588 (0.046)
Average	BMA	0.470 (0.007)	0.283 (0.004)	0.129 (0.002)	0.488 (0.007)	0.301 (0.005)	0.136 (0.002)	0.324 (0.002)
	AIC	1.967 (0.189)	0.300 (0.005)	0.129 (0.002)	2.558 (0.185)	0.327 (0.006)	0.135 (0.002)	1.047 (0.052)
	BIC	2.040 (0.192)	0.309 (0.005)	0.132 (0.002)	2.782 (0.188)	0.339 (0.006)	0.140 (0.002)	1.117 (0.053)
	BF	0.467 (0.007)	0.282 (0.004)	0.129 (0.002)	0.485 (0.007)	0.301 (0.005)	0.135 (0.002)	0.323 (0.002)
	Oracle (Bayes)	0.468 (0.007)	0.275 (0.004)	0.126 (0.002)	0.485 (0.007)	0.294 (0.004)	0.132 (0.002)	0.320 (0.002)
	Oracle (freq)	1.798 (0.290)	0.284 (0.004)	0.126 (0.002)	2.220 (0.216)	0.307 (0.005)	0.132 (0.002)	0.929 (0.065)

Table 7: Error rate / power (SE) for each simulation condition (Part 1) comparing Bayesian model-averaging (BMA) to model selection using information (AIC, BIC), model selection using Bayes factors (BF), and Bayesian or frequentist survival model with the true data generating survival function (Oracle), excluding the parametric models corresponding to the true data generating process. Rows correspond to different simulation conditions according to the true parametric family (Exp = exponential, Weib = Weibull, Log-N = log-normal, Log-L = Log-logistic, Gamma = gamma). Average corresponds to the results aggregated across distributions (last row), log(AF) × N (last column), or the whole simulation (bottom right corner).

**Error**		log(AF) = 0.20			log(AF) = 0.40			Average
		N = 50	N = 200	N = 1000	N = 50	N = 200	N = 1000	
Exp	BMA	0.020 (0.006)	0.008 (0.004)	0.000 (0.000)	0.064 (0.011)	0.098 (0.013)	0.058 (0.010)	0.073 (0.007)
	AIC	0.018 (0.006)	0.002 (0.002)	0.000 (0.000)	0.054 (0.010)	0.068 (0.011)	0.056 (0.010)	0.059 (0.006)
	BIC	0.018 (0.006)	0.002 (0.002)	0.000 (0.000)	0.054 (0.010)	0.068 (0.011)	0.056 (0.010)	0.059 (0.006)
	BF	0.016 (0.006)	0.006 (0.003)	0.000 (0.000)	0.060 (0.011)	0.100 (0.013)	0.062 (0.011)	0.074 (0.007)
	Oracle (Bayes)	0.030 (0.008)	0.010 (0.004)	0.000 (0.000)	0.082 (0.012)	0.104 (0.014)	0.056 (0.010)	0.081 (0.007)
	Oracle (freq)	0.018 (0.006)	0.002 (0.002)	0.000 (0.000)	0.054 (0.010)	0.068 (0.011)	0.056 (0.010)	0.059 (0.006)
Weib	BMA	0.016 (0.006)	0.016 (0.006)	0.000 (0.000)	0.068 (0.011)	0.074 (0.012)	0.058 (0.010)	0.067 (0.006)
	AIC	0.018 (0.006)	0.012 (0.005)	0.000 (0.000)	0.056 (0.010)	0.054 (0.010)	0.060 (0.011)	0.057 (0.006)
	BIC	0.016 (0.006)	0.014 (0.005)	0.000 (0.000)	0.052 (0.010)	0.048 (0.010)	0.060 (0.011)	0.053 (0.006)
	BF	0.018 (0.006)	0.016 (0.006)	0.000 (0.000)	0.066 (0.011)	0.070 (0.011)	0.060 (0.011)	0.065 (0.006)
	Oracle (Bayes)	0.030 (0.008)	0.018 (0.006)	0.000 (0.000)	0.080 (0.012)	0.074 (0.012)	0.052 (0.010)	0.069 (0.007)
	Oracle (freq)	0.016 (0.006)	0.014 (0.005)	0.000 (0.000)	0.052 (0.010)	0.048 (0.010)	0.060 (0.011)	0.053 (0.006)
Log-N	BMA	0.028 (0.007)	0.018 (0.006)	0.000 (0.000)	0.056 (0.010)	0.090 (0.013)	0.040 (0.009)	0.062 (0.006)
	AIC	0.018 (0.006)	0.010 (0.004)	0.000 (0.000)	0.034 (0.008)	0.074 (0.012)	0.038 (0.009)	0.049 (0.006)
	BIC	0.018 (0.006)	0.010 (0.004)	0.000 (0.000)	0.034 (0.008)	0.074 (0.012)	0.038 (0.009)	0.049 (0.006)
	BF	0.032 (0.008)	0.016 (0.006)	0.000 (0.000)	0.056 (0.010)	0.092 (0.013)	0.038 (0.009)	0.062 (0.006)
	Oracle (Bayes)	0.024 (0.007)	0.010 (0.004)	0.000 (0.000)	0.056 (0.010)	0.094 (0.013)	0.034 (0.008)	0.061 (0.006)
	Oracle (freq)	0.018 (0.006)	0.010 (0.004)	0.000 (0.000)	0.034 (0.008)	0.074 (0.012)	0.038 (0.009)	0.049 (0.006)
Log-L	BMA	0.024 (0.007)	0.012 (0.005)	0.000 (0.000)	0.054 (0.010)	0.086 (0.013)	0.050 (0.010)	0.063 (0.006)
	AIC	0.022 (0.007)	0.008 (0.004)	0.000 (0.000)	0.052 (0.010)	0.070 (0.011)	0.054 (0.010)	0.059 (0.006)
	BIC	0.022 (0.007)	0.008 (0.004)	0.000 (0.000)	0.046 (0.009)	0.062 (0.011)	0.052 (0.010)	0.053 (0.006)
	BF	0.028 (0.007)	0.010 (0.004)	0.000 (0.000)	0.056 (0.010)	0.092 (0.013)	0.050 (0.010)	0.066 (0.006)
	Oracle (Bayes)	0.040 (0.009)	0.014 (0.005)	0.000 (0.000)	0.076 (0.012)	0.090 (0.013)	0.046 (0.009)	0.071 (0.007)
	Oracle (freq)	0.022 (0.007)	0.008 (0.004)	0.000 (0.000)	0.046 (0.009)	0.062 (0.011)	0.052 (0.010)	0.053 (0.006)
Gamma	BMA	0.018 (0.006)	0.016 (0.006)	0.000 (0.000)	0.058 (0.010)	0.080 (0.012)	0.056 (0.010)	0.065 (0.006)
	AIC	0.020 (0.006)	0.012 (0.005)	0.000 (0.000)	0.050 (0.010)	0.054 (0.010)	0.056 (0.010)	0.053 (0.006)
	BIC	0.018 (0.006)	0.012 (0.005)	0.000 (0.000)	0.046 (0.009)	0.052 (0.010)	0.056 (0.010)	0.051 (0.006)
	BF	0.020 (0.006)	0.020 (0.006)	0.000 (0.000)	0.062 (0.011)	0.072 (0.012)	0.052 (0.010)	0.062 (0.006)
	Oracle (Bayes)	0.026 (0.007)	0.016 (0.006)	0.000 (0.000)	0.076 (0.012)	0.070 (0.011)	0.048 (0.010)	0.065 (0.006)
	Oracle (freq)	0.018 (0.006)	0.012 (0.005)	0.000 (0.000)	0.046 (0.009)	0.052 (0.010)	0.056 (0.010)	0.051 (0.006)
Average	BMA	0.018 (0.006)	0.016 (0.006)	0.000 (0.000)	0.058 (0.010)	0.080 (0.012)	0.056 (0.010)	0.066 (0.003)
	AIC	0.019 (0.003)	0.009 (0.002)	0.000 (0.000)	0.049 (0.004)	0.064 (0.005)	0.053 (0.004)	0.055 (0.003)
	BIC	0.018 (0.003)	0.009 (0.002)	0.000 (0.000)	0.046 (0.004)	0.061 (0.005)	0.052 (0.004)	0.053 (0.003)
	BF	0.018 (0.006)	0.016 (0.006)	0.000 (0.000)	0.058 (0.010)	0.080 (0.012)	0.056 (0.010)	0.066 (0.003)
	Oracle (Bayes)	0.018 (0.006)	0.016 (0.006)	0.000 (0.000)	0.058 (0.010)	0.080 (0.012)	0.056 (0.010)	0.069 (0.003)
	Oracle (freq)	0.018 (0.003)	0.009 (0.002)	0.000 (0.000)	0.046 (0.004)	0.061 (0.005)	0.052 (0.004)	0.053 (0.003)

Table 8: Error rate / power (SE) for each simulation condition (Part 2) comparing Bayesian model-averaging (BMA) to model selection using information (AIC, BIC), model selection using Bayes factors (BF), and Bayesian or frequentist survival model with the true data generating survival function (Oracle), excluding the parametric models corresponding to the true data generating process. Rows correspond to different simulation conditions according to the true parametric family (Exp = exponential, Weib = Weibull, Log-N = log-normal, Log-L = Log-logistic, Gamma = gamma). Average corresponds to the results aggregated across distributions (last row), log(AF) × N (last column), or the whole simulation (bottom right corner).

**Power**		log(AF) = 0.20			log(AF) = 0.40			Average
		N = 50	N = 200	N = 1000	N = 50	N = 200	N = 1000	
Exp	BMA	0.098 (0.013)	0.202 (0.018)	0.414 (0.022)	0.162 (0.016)	0.394 (0.022)	0.870 (0.015)	0.241 (0.006)
	AIC	0.082 (0.012)	0.138 (0.015)	0.370 (0.022)	0.132 (0.015)	0.324 (0.021)	0.848 (0.016)	0.213 (0.006)
	BIC	0.082 (0.012)	0.138 (0.015)	0.370 (0.022)	0.132 (0.015)	0.324 (0.021)	0.848 (0.016)	0.213 (0.006)
	BF	0.090 (0.013)	0.178 (0.017)	0.420 (0.022)	0.162 (0.016)	0.370 (0.022)	0.872 (0.015)	0.235 (0.006)
	Oracle (Bayes)	0.136 (0.015)	0.206 (0.018)	0.388 (0.022)	0.194 (0.018)	0.404 (0.022)	0.866 (0.015)	0.248 (0.006)
	Oracle (freq)	0.082 (0.012)	0.138 (0.015)	0.370 (0.022)	0.132 (0.015)	0.324 (0.021)	0.848 (0.016)	0.213 (0.006)
Weib	BMA	0.066 (0.011)	0.224 (0.019)	0.518 (0.022)	0.166 (0.017)	0.484 (0.022)	0.962 (0.009)	0.272 (0.007)
	AIC	0.062 (0.011)	0.184 (0.017)	0.522 (0.022)	0.146 (0.016)	0.402 (0.022)	0.946 (0.010)	0.255 (0.006)
	BIC	0.058 (0.010)	0.174 (0.017)	0.522 (0.022)	0.138 (0.015)	0.388 (0.022)	0.946 (0.010)	0.251 (0.006)
	BF	0.066 (0.011)	0.222 (0.019)	0.518 (0.022)	0.170 (0.017)	0.474 (0.022)	0.958 (0.009)	0.271 (0.007)
	Oracle (Bayes)	0.086 (0.013)	0.242 (0.019)	0.504 (0.022)	0.202 (0.018)	0.492 (0.022)	0.954 (0.009)	0.281 (0.007)
	Oracle (freq)	0.058 (0.010)	0.174 (0.017)	0.522 (0.022)	0.138 (0.015)	0.388 (0.022)	0.946 (0.010)	0.251 (0.006)
Log-N	BMA	0.108 (0.014)	0.214 (0.018)	0.426 (0.022)	0.146 (0.016)	0.430 (0.022)	0.900 (0.013)	0.252 (0.006)
	AIC	0.084 (0.012)	0.164 (0.017)	0.440 (0.022)	0.100 (0.013)	0.346 (0.021)	0.898 (0.014)	0.229 (0.006)
	BIC	0.076 (0.012)	0.156 (0.016)	0.436 (0.022)	0.098 (0.013)	0.340 (0.021)	0.898 (0.014)	0.226 (0.006)
	BF	0.110 (0.014)	0.206 (0.018)	0.436 (0.022)	0.142 (0.016)	0.410 (0.022)	0.904 (0.013)	0.251 (0.006)
	Oracle (Bayes)	0.108 (0.014)	0.200 (0.018)	0.448 (0.022)	0.172 (0.017)	0.428 (0.022)	0.918 (0.012)	0.256 (0.007)
	Oracle (freq)	0.076 (0.012)	0.156 (0.016)	0.436 (0.022)	0.098 (0.013)	0.340 (0.021)	0.898 (0.014)	0.226 (0.006)
Log-L	BMA	0.096 (0.013)	0.224 (0.019)	0.508 (0.022)	0.146 (0.016)	0.468 (0.022)	0.916 (0.012)	0.266 (0.007)
	AIC	0.092 (0.013)	0.164 (0.017)	0.510 (0.022)	0.122 (0.015)	0.400 (0.022)	0.916 (0.012)	0.248 (0.006)
	BIC	0.082 (0.012)	0.160 (0.016)	0.506 (0.022)	0.112 (0.014)	0.394 (0.022)	0.916 (0.012)	0.244 (0.006)
	BF	0.098 (0.013)	0.202 (0.018)	0.516 (0.022)	0.142 (0.016)	0.448 (0.022)	0.914 (0.013)	0.262 (0.007)
	Oracle (Bayes)	0.120 (0.015)	0.226 (0.019)	0.500 (0.022)	0.184 (0.017)	0.478 (0.022)	0.918 (0.012)	0.276 (0.007)
	Oracle (freq)	0.082 (0.012)	0.160 (0.016)	0.506 (0.022)	0.112 (0.014)	0.394 (0.022)	0.916 (0.012)	0.244 (0.006)
Gamma	BMA	0.088 (0.013)	0.244 (0.019)	0.552 (0.022)	0.150 (0.016)	0.516 (0.022)	0.956 (0.009)	0.282 (0.007)
	AIC	0.076 (0.012)	0.182 (0.017)	0.556 (0.022)	0.122 (0.015)	0.440 (0.022)	0.956 (0.009)	0.263 (0.007)
	BIC	0.068 (0.011)	0.178 (0.017)	0.554 (0.022)	0.114 (0.014)	0.428 (0.022)	0.954 (0.009)	0.258 (0.007)
	BF	0.090 (0.013)	0.232 (0.019)	0.552 (0.022)	0.148 (0.016)	0.494 (0.022)	0.958 (0.009)	0.279 (0.007)
	Oracle (Bayes)	0.110 (0.014)	0.250 (0.019)	0.532 (0.022)	0.186 (0.017)	0.514 (0.022)	0.958 (0.009)	0.288 (0.007)
	Oracle (freq)	0.068 (0.011)	0.178 (0.017)	0.554 (0.022)	0.114 (0.014)	0.428 (0.022)	0.954 (0.009)	0.258 (0.007)
Average	BMA	0.088 (0.013)	0.244 (0.019)	0.552 (0.022)	0.150 (0.016)	0.516 (0.022)	0.956 (0.009)	0.263 (0.003)
	AIC	0.079 (0.005)	0.166 (0.007)	0.480 (0.010)	0.124 (0.007)	0.382 (0.010)	0.913 (0.006)	0.241 (0.003)
	BIC	0.073 (0.005)	0.161 (0.007)	0.478 (0.010)	0.119 (0.006)	0.375 (0.010)	0.912 (0.006)	0.238 (0.003)
	BF	0.088 (0.013)	0.244 (0.019)	0.552 (0.022)	0.150 (0.016)	0.516 (0.022)	0.956 (0.009)	0.260 (0.003)
	Oracle (Bayes)	0.088 (0.013)	0.244 (0.019)	0.552 (0.022)	0.150 (0.016)	0.516 (0.022)	0.956 (0.009)	0.270 (0.003)
	Oracle (freq)	0.073 (0.005)	0.161 (0.007)	0.478 (0.010)	0.119 (0.006)	0.375 (0.010)	0.912 (0.006)	0.238 (0.003)

Table 9: Confidence intervals coverage (SE) for each simulation condition (Part 1) comparing Bayesian model-averaging (BMA) to model selection using information (AIC, BIC), model selection using Bayes factors (BF), and Bayesian or frequentist survival model with the true data generating survival function (Oracle), excluding the parametric models corresponding to the true data generating process. Rows correspond to different simulation conditions according to the true parametric family (Exp = exponential, Weib = Weibull, Log-N = log-normal, Log-L = Log-logistic, Gamma = gamma). Average corresponds to the results aggregated across distributions (last row), log(AF) × N (last column), or the whole simulation (bottom right corner).

**% CI**		log(AF) = -0.20			log(AF) = 0		
		N = 50	N = 200	N = 1000	N = 50	N = 200	N = 1000
Exp	BMA	0.994 (0.003)	0.978 (0.007)	0.944 (0.010)	0.980 (0.006)	0.954 (0.009)	0.936 (0.011)
	AIC	0.956 (0.009)	0.970 (0.008)	0.936 (0.011)	0.954 (0.009)	0.944 (0.010)	0.934 (0.011)
	BIC	0.956 (0.009)	0.970 (0.008)	0.936 (0.011)	0.954 (0.009)	0.944 (0.010)	0.934 (0.011)
	BF	0.988 (0.005)	0.976 (0.007)	0.940 (0.011)	0.984 (0.006)	0.958 (0.009)	0.932 (0.011)
	Oracle (Bayes)	0.986 (0.005)	0.972 (0.007)	0.936 (0.011)	0.978 (0.007)	0.952 (0.010)	0.934 (0.011)
	Oracle (freq)	0.982 (0.006)	0.968 (0.008)	0.938 (0.011)	0.964 (0.008)	0.946 (0.010)	0.932 (0.011)
Weib	BMA	0.980 (0.006)	0.956 (0.009)	0.958 (0.009)	0.982 (0.006)	0.960 (0.009)	0.942 (0.010)
	AIC	0.966 (0.008)	0.948 (0.010)	0.952 (0.010)	0.938 (0.011)	0.954 (0.009)	0.938 (0.011)
	BIC	0.970 (0.008)	0.952 (0.010)	0.954 (0.009)	0.956 (0.009)	0.958 (0.009)	0.940 (0.011)
	BF	0.986 (0.005)	0.958 (0.009)	0.956 (0.009)	0.986 (0.005)	0.962 (0.009)	0.942 (0.010)
	Oracle (Bayes)	0.976 (0.007)	0.956 (0.009)	0.952 (0.010)	0.980 (0.006)	0.954 (0.009)	0.934 (0.011)
	Oracle (freq)	0.972 (0.007)	0.944 (0.010)	0.952 (0.010)	0.976 (0.007)	0.956 (0.009)	0.938 (0.011)
Log-N	BMA	0.972 (0.007)	0.956 (0.009)	0.956 (0.009)	0.984 (0.006)	0.956 (0.009)	0.960 (0.009)
	AIC	0.966 (0.008)	0.948 (0.010)	0.948 (0.010)	0.976 (0.007)	0.942 (0.010)	0.962 (0.009)
	BIC	0.966 (0.008)	0.946 (0.010)	0.948 (0.010)	0.978 (0.007)	0.942 (0.010)	0.962 (0.009)
	BF	0.972 (0.007)	0.956 (0.009)	0.954 (0.009)	0.986 (0.005)	0.948 (0.010)	0.960 (0.009)
	Oracle (Bayes)	0.974 (0.007)	0.970 (0.008)	0.964 (0.008)	0.988 (0.005)	0.962 (0.009)	0.956 (0.009)
	Oracle (freq)	0.964 (0.008)	0.952 (0.010)	0.960 (0.009)	0.964 (0.008)	0.948 (0.010)	0.958 (0.009)
Log-L	BMA	0.968 (0.008)	0.950 (0.010)	0.946 (0.010)	0.978 (0.007)	0.956 (0.009)	0.958 (0.009)
	AIC	0.952 (0.010)	0.942 (0.010)	0.942 (0.010)	0.950 (0.010)	0.950 (0.010)	0.958 (0.009)
	BIC	0.954 (0.009)	0.944 (0.010)	0.940 (0.011)	0.956 (0.009)	0.952 (0.010)	0.958 (0.009)
	BF	0.968 (0.008)	0.956 (0.009)	0.942 (0.010)	0.976 (0.007)	0.952 (0.010)	0.954 (0.009)
	Oracle (Bayes)	0.974 (0.007)	0.952 (0.010)	0.944 (0.010)	0.976 (0.007)	0.956 (0.009)	0.952 (0.010)
	Oracle (freq)	0.958 (0.009)	0.946 (0.010)	0.942 (0.010)	0.964 (0.008)	0.954 (0.009)	0.952 (0.010)
Gamma	BMA	0.982 (0.006)	0.950 (0.010)	0.956 (0.009)	0.980 (0.006)	0.962 (0.009)	0.944 (0.010)
	AIC	0.968 (0.008)	0.932 (0.011)	0.950 (0.010)	0.962 (0.009)	0.954 (0.009)	0.942 (0.010)
	BIC	0.972 (0.007)	0.938 (0.011)	0.948 (0.010)	0.968 (0.008)	0.952 (0.010)	0.942 (0.010)
	BF	0.982 (0.006)	0.950 (0.010)	0.954 (0.009)	0.982 (0.006)	0.956 (0.009)	0.940 (0.011)
	Oracle (Bayes)	0.982 (0.006)	0.946 (0.010)	0.946 (0.010)	0.978 (0.007)	0.956 (0.009)	0.940 (0.011)
	Oracle (freq)	0.976 (0.007)	0.944 (0.010)	0.948 (0.010)	0.982 (0.006)	0.960 (0.009)	0.942 (0.010)
Average	BMA	0.979 (0.003)	0.958 (0.004)	0.952 (0.004)	0.981 (0.003)	0.958 (0.004)	0.948 (0.004)
	AIC	0.962 (0.004)	0.948 (0.004)	0.946 (0.005)	0.956 (0.004)	0.949 (0.004)	0.947 (0.004)
	BIC	0.964 (0.004)	0.950 (0.004)	0.945 (0.005)	0.962 (0.004)	0.950 (0.004)	0.947 (0.004)
	BF	0.979 (0.003)	0.959 (0.004)	0.949 (0.004)	0.983 (0.003)	0.955 (0.004)	0.946 (0.005)
	Oracle (Bayes)	0.978 (0.003)	0.959 (0.004)	0.948 (0.004)	0.980 (0.003)	0.956 (0.004)	0.943 (0.005)
	Oracle (freq)	0.970 (0.003)	0.951 (0.004)	0.948 (0.004)	0.970 (0.003)	0.953 (0.004)	0.944 (0.005)

Table 10: Bias (SE) for each simulation condition (Part 2) comparing Bayesian model-averaging (BMA) to model selection using information (AIC, BIC), model selection using Bayes factors (BF), and Bayesian or frequentist survival model with the true data generating survival function (Oracle), excluding the parametric models corresponding to the true data generating process. Rows correspond to different simulation conditions according to the true parametric family (Exp = exponential, Weib = Weibull, Log-N = log-normal, Log-L = Log-logistic, Gamma = gamma). Average corresponds to the results aggregated across distributions (last row), log(AF) × N (last column), or the whole simulation (bottom right corner).

**% CI**		log(AF) = 0.20			log(AF) = 0.40			Average
		N = 50	N = 200	N = 1000	N = 50	N = 200	N = 1000	
Exp	BMA	0.982 (0.006)	0.948 (0.010)	0.954 (0.009)	0.984 (0.006)	0.962 (0.009)	0.950 (0.010)	0.964 (0.002)
	AIC	0.974 (0.007)	0.944 (0.010)	0.956 (0.009)	0.970 (0.008)	0.940 (0.011)	0.942 (0.010)	0.952 (0.003)
	BIC	0.974 (0.007)	0.944 (0.010)	0.956 (0.009)	0.970 (0.008)	0.940 (0.011)	0.942 (0.010)	0.952 (0.003)
	BF	0.984 (0.006)	0.954 (0.009)	0.958 (0.009)	0.984 (0.006)	0.956 (0.009)	0.946 (0.010)	0.963 (0.002)
	Oracle (Bayes)	0.974 (0.007)	0.946 (0.010)	0.962 (0.009)	0.974 (0.007)	0.958 (0.009)	0.956 (0.009)	0.961 (0.003)
	Oracle (freq)	0.970 (0.008)	0.950 (0.010)	0.960 (0.009)	0.970 (0.008)	0.958 (0.009)	0.956 (0.009)	0.958 (0.003)
Weib	BMA	0.988 (0.005)	0.974 (0.007)	0.948 (0.010)	0.980 (0.006)	0.966 (0.008)	0.964 (0.008)	0.967 (0.002)
	AIC	0.974 (0.007)	0.958 (0.009)	0.942 (0.010)	0.978 (0.007)	0.962 (0.009)	0.964 (0.008)	0.956 (0.003)
	BIC	0.974 (0.007)	0.962 (0.009)	0.942 (0.010)	0.978 (0.007)	0.954 (0.009)	0.960 (0.009)	0.958 (0.003)
	BF	0.984 (0.006)	0.972 (0.007)	0.940 (0.011)	0.986 (0.005)	0.966 (0.008)	0.964 (0.008)	0.967 (0.002)
	Oracle (Bayes)	0.988 (0.005)	0.974 (0.007)	0.940 (0.011)	0.980 (0.006)	0.962 (0.009)	0.964 (0.008)	0.963 (0.002)
	Oracle (freq)	0.992 (0.004)	0.970 (0.008)	0.946 (0.010)	0.978 (0.007)	0.968 (0.008)	0.964 (0.008)	0.963 (0.002)
Log-N	BMA	0.984 (0.006)	0.966 (0.008)	0.974 (0.007)	0.978 (0.007)	0.966 (0.008)	0.934 (0.011)	0.966 (0.002)
	AIC	0.972 (0.007)	0.964 (0.008)	0.974 (0.007)	0.960 (0.009)	0.948 (0.010)	0.924 (0.012)	0.957 (0.003)
	BIC	0.974 (0.007)	0.960 (0.009)	0.974 (0.007)	0.966 (0.008)	0.956 (0.009)	0.924 (0.012)	0.958 (0.003)
	BF	0.986 (0.005)	0.968 (0.008)	0.974 (0.007)	0.974 (0.007)	0.964 (0.008)	0.932 (0.011)	0.965 (0.002)
	Oracle (Bayes)	0.990 (0.004)	0.980 (0.006)	0.978 (0.007)	0.982 (0.006)	0.964 (0.008)	0.944 (0.010)	0.971 (0.002)
	Oracle (freq)	0.958 (0.009)	0.966 (0.008)	0.976 (0.007)	0.950 (0.010)	0.952 (0.010)	0.940 (0.011)	0.957 (0.003)
Log-L	BMA	0.978 (0.007)	0.966 (0.008)	0.944 (0.010)	0.984 (0.006)	0.958 (0.009)	0.954 (0.009)	0.962 (0.002)
	AIC	0.964 (0.008)	0.950 (0.010)	0.942 (0.010)	0.962 (0.009)	0.944 (0.010)	0.944 (0.010)	0.950 (0.003)
	BIC	0.966 (0.008)	0.954 (0.009)	0.942 (0.010)	0.964 (0.008)	0.944 (0.010)	0.948 (0.010)	0.952 (0.003)
	BF	0.980 (0.006)	0.972 (0.007)	0.942 (0.010)	0.978 (0.007)	0.952 (0.010)	0.948 (0.010)	0.960 (0.003)
	Oracle (Bayes)	0.978 (0.007)	0.966 (0.008)	0.958 (0.009)	0.984 (0.006)	0.962 (0.009)	0.950 (0.010)	0.963 (0.002)
	Oracle (freq)	0.970 (0.008)	0.958 (0.009)	0.958 (0.009)	0.970 (0.008)	0.960 (0.009)	0.948 (0.010)	0.957 (0.003)
Gamma	BMA	0.984 (0.006)	0.952 (0.010)	0.948 (0.010)	0.982 (0.006)	0.970 (0.008)	0.966 (0.008)	0.965 (0.002)
	AIC	0.958 (0.009)	0.940 (0.011)	0.954 (0.009)	0.964 (0.008)	0.956 (0.009)	0.964 (0.008)	0.954 (0.003)
	BIC	0.970 (0.008)	0.948 (0.010)	0.946 (0.010)	0.968 (0.008)	0.958 (0.009)	0.958 (0.009)	0.956 (0.003)
	BF	0.984 (0.006)	0.952 (0.010)	0.942 (0.010)	0.982 (0.006)	0.966 (0.008)	0.964 (0.008)	0.963 (0.002)
	Oracle (Bayes)	0.974 (0.007)	0.948 (0.010)	0.944 (0.010)	0.974 (0.007)	0.968 (0.008)	0.968 (0.008)	0.960 (0.003)
	Oracle (freq)	0.972 (0.007)	0.944 (0.010)	0.950 (0.010)	0.976 (0.007)	0.966 (0.008)	0.966 (0.008)	0.961 (0.003)
Average	BMA	0.983 (0.003)	0.961 (0.004)	0.954 (0.004)	0.982 (0.003)	0.964 (0.004)	0.954 (0.004)	0.964 (0.001)
	AIC	0.968 (0.003)	0.951 (0.004)	0.954 (0.004)	0.967 (0.004)	0.950 (0.004)	0.948 (0.004)	0.954 (0.001)
	BIC	0.972 (0.003)	0.954 (0.004)	0.952 (0.004)	0.969 (0.003)	0.950 (0.004)	0.946 (0.005)	0.955 (0.001)
	BF	0.984 (0.003)	0.964 (0.004)	0.951 (0.004)	0.981 (0.003)	0.961 (0.004)	0.951 (0.004)	0.964 (0.001)
	Oracle (Bayes)	0.981 (0.003)	0.963 (0.004)	0.956 (0.004)	0.979 (0.003)	0.963 (0.004)	0.956 (0.004)	0.964 (0.001)
	Oracle (freq)	0.972 (0.003)	0.958 (0.004)	0.958 (0.004)	0.969 (0.003)	0.961 (0.004)	0.955 (0.004)	0.959 (0.001)

Table 11: Bias (SE) of predicted mean survival in 20 years for each simulation condition (Part 1) comparing Bayesian model-averaging (BMA) to model selection using information (AIC, BIC), model selection using Bayes factors (BF), and Bayesian or frequentist survival model with the true data generating survival function (Oracle), excluding the parametric models corresponding to the true data generating process. Rows correspond to different simulation conditions according to the true parametric family (Exp = exponential, Weib = Weibull, Log-N = log-normal, Log-L = Log-logistic, Gamma = gamma). Average corresponds to the results aggregated across distributions (last row), log(AF) × N (last column), or the whole simulation (bottom right corner).

**Bias**		log(AF) = -0.20			log(AF) = 0		
		N = 50	N = 200	N = 1000	N = 50	N = 200	N = 1000
Exp	BMA	0.162 (0.003)	0.100 (0.002)	0.048 (0.002)	0.181 (0.003)	0.113 (0.002)	0.053 (0.002)
	BF	0.194 (0.004)	0.102 (0.003)	0.048 (0.002)	0.219 (0.004)	0.122 (0.003)	0.052 (0.002)
	AIC	0.128 (0.006)	0.076 (0.004)	0.041 (0.002)	0.140 (0.006)	0.088 (0.004)	0.045 (0.003)
	BIC	0.128 (0.006)	0.076 (0.004)	0.041 (0.002)	0.140 (0.006)	0.088 (0.004)	0.045 (0.003)
	Oracle (Bayes)	0.064 (0.003)	0.016 (0.002)	0.003 (0.001)	0.085 (0.004)	0.023 (0.002)	0.005 (0.001)
	Oracle (freq)	0.039 (0.004)	0.006 (0.002)	0.001 (0.001)	0.059 (0.005)	0.012 (0.002)	0.003 (0.001)
Weib	BMA	0.184 (0.003)	0.111 (0.002)	0.069 (0.001)	0.198 (0.003)	0.132 (0.002)	0.085 (0.001)
	BF	0.193 (0.004)	0.105 (0.003)	0.059 (0.002)	0.212 (0.005)	0.125 (0.003)	0.077 (0.002)
	AIC	0.095 (0.004)	0.075 (0.003)	0.045 (0.002)	0.101 (0.004)	0.088 (0.003)	0.059 (0.002)
	BIC	0.093 (0.004)	0.066 (0.002)	0.049 (0.002)	0.101 (0.004)	0.081 (0.002)	0.064 (0.002)
	Oracle (Bayes)	0.141 (0.003)	0.051 (0.002)	0.013 (0.001)	0.156 (0.004)	0.069 (0.002)	0.017 (0.001)
	Oracle (freq)	0.043 (0.004)	0.009 (0.002)	0.002 (0.001)	0.047 (0.004)	0.015 (0.002)	0.003 (0.001)
Log-N	BMA	-0.038 (0.003)	-0.097 (0.001)	-0.088 (0.001)	-0.031 (0.003)	-0.100 (0.002)	-0.103 (0.001)
	BF	-0.048 (0.003)	-0.078 (0.002)	-0.054 (0.001)	-0.040 (0.004)	-0.078 (0.002)	-0.062 (0.001)
	AIC	-0.100 (0.004)	-0.100 (0.002)	-0.063 (0.001)	-0.106 (0.004)	-0.107 (0.002)	-0.074 (0.001)
	BIC	-0.089 (0.004)	-0.116 (0.002)	-0.068 (0.001)	-0.089 (0.004)	-0.116 (0.002)	-0.077 (0.001)
	Oracle (Bayes)	0.102 (0.003)	0.043 (0.002)	0.009 (0.001)	0.111 (0.003)	0.045 (0.002)	0.008 (0.001)
	Oracle (freq)	-0.005 (0.005)	0.001 (0.002)	-0.002 (0.001)	-0.003 (0.005)	-0.003 (0.002)	-0.003 (0.001)
Log-L	BMA	0.041 (0.003)	-0.021 (0.002)	-0.078 (0.002)	0.050 (0.003)	-0.014 (0.002)	-0.078 (0.002)
	BF	0.083 (0.004)	0.003 (0.003)	-0.074 (0.002)	0.093 (0.005)	0.011 (0.003)	-0.070 (0.003)
	AIC	-0.032 (0.004)	-0.040 (0.003)	-0.086 (0.002)	-0.033 (0.005)	-0.042 (0.003)	-0.088 (0.003)
	BIC	-0.029 (0.004)	-0.050 (0.002)	-0.083 (0.002)	-0.030 (0.005)	-0.048 (0.002)	-0.084 (0.002)
	Oracle (Bayes)	0.108 (0.003)	0.043 (0.002)	0.007 (0.001)	0.116 (0.003)	0.047 (0.002)	0.008 (0.001)
	Oracle (freq)	0.015 (0.004)	0.005 (0.002)	-0.001 (0.001)	0.014 (0.005)	0.003 (0.002)	-0.002 (0.001)
Gamma	BMA	0.179 (0.003)	0.104 (0.002)	0.049 (0.001)	0.196 (0.003)	0.119 (0.002)	0.066 (0.002)
	BF	0.192 (0.004)	0.100 (0.003)	0.041 (0.002)	0.214 (0.004)	0.125 (0.003)	0.059 (0.003)
	AIC	0.085 (0.004)	0.065 (0.003)	0.030 (0.002)	0.093 (0.005)	0.072 (0.003)	0.043 (0.003)
	BIC	0.085 (0.004)	0.059 (0.002)	0.031 (0.002)	0.095 (0.005)	0.066 (0.002)	0.047 (0.002)
	Oracle (Bayes)	0.128 (0.003)	0.042 (0.002)	0.006 (0.001)	0.144 (0.004)	0.048 (0.002)	0.012 (0.001)
	Oracle (freq)	0.043 (0.004)	0.011 (0.001)	0.000 (0.001)	0.044 (0.004)	0.009 (0.002)	0.003 (0.001)
Average	BMA	0.106 (0.002)	0.040 (0.001)	0.000 (0.001)	0.119 (0.002)	0.050 (0.002)	0.005 (0.001)
	BF	0.123 (0.002)	0.046 (0.002)	0.004 (0.001)	0.139 (0.002)	0.061 (0.002)	0.011 (0.001)
	AIC	0.035 (0.002)	0.015 (0.002)	-0.007 (0.001)	0.039 (0.003)	0.020 (0.002)	-0.003 (0.001)
	BIC	0.037 (0.002)	0.007 (0.002)	-0.006 (0.001)	0.043 (0.002)	0.014 (0.002)	-0.001 (0.001)
	Oracle (Bayes)	0.108 (0.002)	0.039 (0.001)	0.008 (0.000)	0.122 (0.002)	0.047 (0.001)	0.010 (0.000)
	Oracle (freq)	0.027 (0.002)	0.006 (0.001)	0.000 (0.000)	0.032 (0.002)	0.007 (0.001)	0.001 (0.000)

Table 12: Bias (SE) of predicted mean survival in 20 years for each simulation condition (Part 2) comparing Bayesian model-averaging (BMA) to model selection using information (AIC, BIC), model selection using Bayes factors (BF), and Bayesian or frequentist survival model with the true data generating survival function (Oracle), excluding the parametric models corresponding to the true data generating process. Rows correspond to different simulation conditions according to the true parametric family (Exp = exponential, Weib = Weibull, Log-N = log-normal, Log-L = Log-logistic, Gamma = gamma). Average corresponds to the results aggregated across distributions (last row), log(AF) × N (last column), or the whole simulation (bottom right corner).

**Bias**		log(AF) = 0.20			log(AF) = 0.40			Average
		N = 50	N = 200	N = 1000	N = 50	N = 200	N = 1000	
Exp	BMA	0.164 (0.004)	0.108 (0.003)	0.055 (0.002)	0.165 (0.004)	0.105 (0.003)	0.058 (0.002)	0.110 (0.001)
	BF	0.200 (0.004)	0.118 (0.004)	0.050 (0.003)	0.203 (0.004)	0.119 (0.004)	0.049 (0.002)	0.123 (0.001)
	AIC	0.126 (0.007)	0.084 (0.004)	0.048 (0.003)	0.130 (0.007)	0.085 (0.004)	0.050 (0.003)	0.087 (0.001)
	BIC	0.126 (0.007)	0.084 (0.004)	0.048 (0.003)	0.130 (0.007)	0.085 (0.004)	0.050 (0.003)	0.087 (0.001)
	Oracle (Bayes)	0.074 (0.004)	0.023 (0.002)	0.004 (0.001)	0.077 (0.004)	0.018 (0.002)	0.004 (0.001)	0.033 (0.001)
	Oracle (freq)	0.051 (0.005)	0.012 (0.002)	0.001 (0.001)	0.059 (0.006)	0.007 (0.002)	0.002 (0.001)	0.021 (0.001)
Weib	BMA	0.208 (0.003)	0.143 (0.002)	0.092 (0.001)	0.221 (0.004)	0.152 (0.003)	0.095 (0.002)	0.141 (0.001)
	BF	0.220 (0.005)	0.140 (0.003)	0.081 (0.002)	0.239 (0.005)	0.146 (0.003)	0.081 (0.002)	0.140 (0.001)
	AIC	0.103 (0.005)	0.099 (0.003)	0.060 (0.002)	0.121 (0.006)	0.102 (0.003)	0.060 (0.002)	0.084 (0.001)
	BIC	0.108 (0.005)	0.090 (0.002)	0.069 (0.002)	0.128 (0.006)	0.103 (0.003)	0.072 (0.002)	0.085 (0.001)
	Oracle (Bayes)	0.168 (0.004)	0.079 (0.002)	0.019 (0.001)	0.185 (0.004)	0.090 (0.003)	0.019 (0.001)	0.084 (0.001)
	Oracle (freq)	0.042 (0.005)	0.016 (0.002)	0.001 (0.001)	0.062 (0.006)	0.019 (0.003)	0.000 (0.001)	0.022 (0.001)
Log-N	BMA	-0.030 (0.004)	-0.102 (0.002)	-0.114 (0.001)	-0.019 (0.004)	-0.101 (0.002)	-0.122 (0.001)	-0.079 (0.001)
	BF	-0.036 (0.004)	-0.078 (0.002)	-0.067 (0.001)	-0.026 (0.004)	-0.078 (0.002)	-0.075 (0.001)	-0.060 (0.001)
	AIC	-0.105 (0.005)	-0.119 (0.002)	-0.080 (0.001)	-0.102 (0.006)	-0.121 (0.003)	-0.092 (0.001)	-0.098 (0.001)
	BIC	-0.084 (0.005)	-0.113 (0.002)	-0.085 (0.001)	-0.068 (0.006)	-0.111 (0.003)	-0.094 (0.001)	-0.093 (0.001)
	Oracle (Bayes)	0.108 (0.003)	0.047 (0.002)	0.012 (0.001)	0.114 (0.003)	0.046 (0.002)	0.010 (0.001)	0.055 (0.001)
	Oracle (freq)	-0.018 (0.005)	-0.005 (0.003)	-0.001 (0.001)	-0.011 (0.006)	-0.008 (0.003)	-0.003 (0.001)	-0.005 (0.001)
Log-L	BMA	0.063 (0.004)	-0.003 (0.002)	-0.070 (0.002)	0.080 (0.004)	0.000 (0.003)	-0.057 (0.002)	-0.007 (0.001)
	BF	0.109 (0.005)	0.029 (0.003)	-0.063 (0.003)	0.127 (0.004)	0.028 (0.004)	-0.050 (0.003)	0.019 (0.001)
	AIC	-0.024 (0.005)	-0.029 (0.003)	-0.086 (0.003)	-0.008 (0.006)	-0.044 (0.004)	-0.075 (0.003)	-0.049 (0.001)
	BIC	-0.017 (0.005)	-0.042 (0.002)	-0.075 (0.003)	0.008 (0.006)	-0.036 (0.003)	-0.064 (0.003)	-0.046 (0.001)
	Oracle (Bayes)	0.126 (0.004)	0.054 (0.002)	0.011 (0.001)	0.137 (0.004)	0.052 (0.002)	0.014 (0.001)	0.060 (0.001)
	Oracle (freq)	0.017 (0.005)	0.007 (0.003)	-0.001 (0.001)	0.021 (0.006)	-0.001 (0.003)	0.001 (0.001)	0.006 (0.001)
Gamma	BMA	0.207 (0.003)	0.130 (0.002)	0.069 (0.002)	0.206 (0.004)	0.137 (0.002)	0.079 (0.002)	0.129 (0.001)
	BF	0.227 (0.005)	0.139 (0.003)	0.063 (0.003)	0.227 (0.005)	0.142 (0.003)	0.072 (0.003)	0.133 (0.001)
	AIC	0.103 (0.005)	0.079 (0.003)	0.040 (0.003)	0.108 (0.006)	0.080 (0.003)	0.050 (0.003)	0.071 (0.001)
	BIC	0.110 (0.005)	0.077 (0.002)	0.047 (0.002)	0.117 (0.006)	0.086 (0.003)	0.056 (0.003)	0.073 (0.001)
	Oracle (Bayes)	0.158 (0.004)	0.056 (0.002)	0.011 (0.001)	0.160 (0.004)	0.062 (0.003)	0.014 (0.001)	0.070 (0.001)
	Oracle (freq)	0.050 (0.005)	0.009 (0.002)	0.000 (0.001)	0.048 (0.006)	0.010 (0.003)	0.002 (0.001)	0.019 (0.001)
Average	BMA	0.122 (0.002)	0.056 (0.002)	0.006 (0.001)	0.131 (0.002)	0.058 (0.002)	0.011 (0.001)	0.059 (0.001)
	BF	0.144 (0.002)	0.069 (0.002)	0.013 (0.001)	0.154 (0.002)	0.071 (0.002)	0.016 (0.001)	0.071 (0.001)
	AIC	0.040 (0.003)	0.023 (0.002)	-0.004 (0.001)	0.050 (0.003)	0.021 (0.002)	-0.001 (0.001)	0.019 (0.001)
	BIC	0.048 (0.003)	0.019 (0.002)	0.001 (0.001)	0.063 (0.003)	0.025 (0.002)	0.004 (0.001)	0.021 (0.001)
	Oracle (Bayes)	0.127 (0.002)	0.052 (0.001)	0.011 (0.000)	0.135 (0.002)	0.054 (0.001)	0.012 (0.001)	0.060 (0.000)
	Oracle (freq)	0.028 (0.002)	0.008 (0.001)	0.000 (0.000)	0.036 (0.003)	0.005 (0.001)	0.000 (0.001)	0.013 (0.000)

Table 13: RMSE (SE) of predicted mean survival in 20 years for each simulation condition (Part 1) comparing Bayesian model-averaging (BMA) to model selection using information (AIC, BIC), model selection using Bayes factors (BF), and Bayesian or frequentist survival model with the true data generating survival function (Oracle), excluding the parametric models corresponding to the true data generating process. Rows correspond to different simulation conditions according to the true parametric family (Exp = exponential, Weib = Weibull, Log-N = log-normal, Log-L = Log-logistic, Gamma = gamma). Average corresponds to the results aggregated across distributions (last row), log(AF) × N (last column), or the whole simulation (bottom right corner).

**RMSE**		log(AF) = -0.20			log(AF) = 0		
		N = 50	N = 200	N = 1000	N = 50	N = 200	N = 1000
Exp	BMA	0.191 (0.003)	0.122 (0.002)	0.068 (0.002)	0.210 (0.003)	0.134 (0.002)	0.073 (0.002)
	BF	0.233 (0.004)	0.146 (0.003)	0.087 (0.002)	0.254 (0.004)	0.164 (0.003)	0.093 (0.003)
	AIC	0.217 (0.006)	0.136 (0.003)	0.085 (0.002)	0.235 (0.006)	0.149 (0.003)	0.093 (0.002)
	BIC	0.217 (0.006)	0.136 (0.003)	0.085 (0.002)	0.235 (0.006)	0.149 (0.003)	0.093 (0.002)
	Oracle (Bayes)	0.123 (0.004)	0.050 (0.001)	0.022 (0.001)	0.146 (0.004)	0.062 (0.002)	0.027 (0.001)
	Oracle (freq)	0.136 (0.007)	0.049 (0.001)	0.022 (0.001)	0.158 (0.007)	0.061 (0.002)	0.027 (0.001)
Weib	BMA	0.206 (0.003)	0.124 (0.002)	0.077 (0.001)	0.223 (0.003)	0.145 (0.002)	0.093 (0.001)
	BF	0.235 (0.004)	0.135 (0.003)	0.084 (0.002)	0.258 (0.004)	0.158 (0.003)	0.103 (0.002)
	AIC	0.153 (0.005)	0.109 (0.003)	0.072 (0.002)	0.169 (0.006)	0.125 (0.003)	0.089 (0.002)
	BIC	0.151 (0.006)	0.088 (0.002)	0.069 (0.002)	0.169 (0.006)	0.102 (0.002)	0.085 (0.002)
	Oracle (Bayes)	0.176 (0.004)	0.074 (0.002)	0.026 (0.001)	0.197 (0.004)	0.096 (0.002)	0.033 (0.001)
	Oracle (freq)	0.124 (0.006)	0.049 (0.002)	0.021 (0.001)	0.147 (0.006)	0.066 (0.003)	0.027 (0.001)
Log-N	BMA	0.105 (0.002)	0.107 (0.001)	0.093 (0.001)	0.109 (0.003)	0.112 (0.001)	0.106 (0.001)
	BF	0.120 (0.002)	0.099 (0.002)	0.061 (0.001)	0.125 (0.003)	0.100 (0.002)	0.069 (0.001)
	AIC	0.167 (0.004)	0.118 (0.002)	0.070 (0.001)	0.175 (0.003)	0.125 (0.002)	0.081 (0.001)
	BIC	0.165 (0.004)	0.129 (0.002)	0.074 (0.001)	0.168 (0.003)	0.132 (0.002)	0.083 (0.001)
	Oracle (Bayes)	0.143 (0.003)	0.076 (0.002)	0.032 (0.001)	0.149 (0.003)	0.083 (0.002)	0.033 (0.001)
	Oracle (freq)	0.143 (0.005)	0.069 (0.002)	0.032 (0.001)	0.151 (0.004)	0.077 (0.002)	0.032 (0.001)
Log-L	BMA	0.107 (0.003)	0.066 (0.001)	0.095 (0.001)	0.117 (0.004)	0.066 (0.001)	0.098 (0.001)
	BF	0.158 (0.003)	0.096 (0.002)	0.104 (0.001)	0.173 (0.004)	0.106 (0.002)	0.111 (0.001)
	AIC	0.139 (0.005)	0.102 (0.002)	0.111 (0.001)	0.147 (0.005)	0.109 (0.002)	0.120 (0.001)
	BIC	0.138 (0.005)	0.083 (0.002)	0.105 (0.001)	0.147 (0.006)	0.086 (0.002)	0.113 (0.001)
	Oracle (Bayes)	0.147 (0.003)	0.071 (0.002)	0.026 (0.001)	0.158 (0.004)	0.077 (0.002)	0.030 (0.001)
	Oracle (freq)	0.134 (0.005)	0.058 (0.002)	0.025 (0.001)	0.143 (0.005)	0.064 (0.002)	0.029 (0.001)
Gamma	BMA	0.202 (0.003)	0.118 (0.002)	0.064 (0.001)	0.221 (0.004)	0.133 (0.002)	0.082 (0.002)
	BF	0.233 (0.004)	0.131 (0.003)	0.077 (0.002)	0.257 (0.004)	0.159 (0.003)	0.099 (0.002)
	AIC	0.156 (0.006)	0.106 (0.003)	0.073 (0.002)	0.173 (0.006)	0.122 (0.003)	0.091 (0.002)
	BIC	0.148 (0.006)	0.085 (0.002)	0.069 (0.002)	0.174 (0.006)	0.095 (0.002)	0.087 (0.002)
	Oracle (Bayes)	0.167 (0.004)	0.067 (0.002)	0.020 (0.001)	0.187 (0.004)	0.078 (0.002)	0.027 (0.001)
	Oracle (freq)	0.131 (0.007)	0.047 (0.002)	0.018 (0.001)	0.144 (0.007)	0.056 (0.002)	0.023 (0.001)
Average	BMA	0.169 (0.002)	0.110 (0.001)	0.081 (0.001)	0.183 (0.002)	0.121 (0.001)	0.091 (0.001)
	BF	0.202 (0.002)	0.123 (0.001)	0.084 (0.001)	0.220 (0.002)	0.140 (0.001)	0.096 (0.001)
	AIC	0.169 (0.002)	0.115 (0.001)	0.084 (0.001)	0.182 (0.003)	0.127 (0.001)	0.096 (0.001)
	BIC	0.166 (0.002)	0.107 (0.001)	0.082 (0.001)	0.181 (0.003)	0.115 (0.001)	0.093 (0.001)
	Oracle (Bayes)	0.152 (0.002)	0.068 (0.001)	0.026 (0.000)	0.169 (0.002)	0.080 (0.001)	0.030 (0.000)
	Oracle (freq)	0.134 (0.003)	0.055 (0.001)	0.024 (0.000)	0.149 (0.003)	0.065 (0.001)	0.028 (0.000)

Table 14: RMSE (SE) of predicted mean survival in 20 years for each simulation condition (Part 2) comparing Bayesian model-averaging (BMA) to model selection using information (AIC, BIC), model selection using Bayes factors (BF), and Bayesian or frequentist survival model with the true data generating survival function (Oracle), excluding the parametric models corresponding to the true data generating process. Rows correspond to different simulation conditions according to the true parametric family (Exp = exponential, Weib = Weibull, Log-N = log-normal, Log-L = Log-logistic, Gamma = gamma). Average corresponds to the results aggregated across distributions (last row), log(AF) × N (last column), or the whole simulation (bottom right corner).

**RMSE**		log(AF) = 0.20			log(AF) = 0.40			Average
		N = 50	N = 200	N = 1000	N = 50	N = 200	N = 1000	
Exp	BMA	0.199 (0.004)	0.137 (0.003)	0.080 (0.002)	0.205 (0.004)	0.141 (0.003)	0.085 (0.002)	0.146 (0.001)
	BF	0.241 (0.004)	0.168 (0.003)	0.095 (0.003)	0.244 (0.004)	0.171 (0.003)	0.092 (0.002)	0.177 (0.001)
	AIC	0.241 (0.007)	0.160 (0.003)	0.099 (0.002)	0.249 (0.006)	0.164 (0.003)	0.099 (0.002)	0.171 (0.002)
	BIC	0.241 (0.007)	0.160 (0.003)	0.099 (0.002)	0.249 (0.006)	0.164 (0.003)	0.099 (0.002)	0.171 (0.002)
	Oracle (Bayes)	0.147 (0.004)	0.071 (0.002)	0.030 (0.001)	0.158 (0.004)	0.077 (0.002)	0.034 (0.001)	0.093 (0.001)
	Oracle (freq)	0.175 (0.007)	0.071 (0.002)	0.029 (0.001)	0.191 (0.007)	0.079 (0.002)	0.034 (0.001)	0.104 (0.002)
Weib	BMA	0.232 (0.003)	0.158 (0.002)	0.102 (0.002)	0.250 (0.004)	0.172 (0.003)	0.108 (0.002)	0.167 (0.001)
	BF	0.263 (0.004)	0.171 (0.003)	0.107 (0.002)	0.281 (0.004)	0.179 (0.003)	0.109 (0.002)	0.186 (0.001)
	AIC	0.183 (0.007)	0.138 (0.003)	0.092 (0.002)	0.213 (0.007)	0.145 (0.004)	0.095 (0.002)	0.138 (0.002)
	BIC	0.185 (0.007)	0.113 (0.002)	0.093 (0.002)	0.219 (0.007)	0.135 (0.003)	0.098 (0.002)	0.133 (0.002)
	Oracle (Bayes)	0.208 (0.004)	0.110 (0.003)	0.040 (0.001)	0.229 (0.004)	0.128 (0.003)	0.047 (0.002)	0.133 (0.001)
	Oracle (freq)	0.164 (0.008)	0.080 (0.003)	0.034 (0.001)	0.207 (0.007)	0.100 (0.003)	0.043 (0.001)	0.106 (0.002)
Log-N	BMA	0.118 (0.003)	0.117 (0.001)	0.120 (0.001)	0.124 (0.003)	0.124 (0.002)	0.128 (0.001)	0.114 (0.001)
	BF	0.131 (0.003)	0.103 (0.002)	0.076 (0.001)	0.135 (0.003)	0.107 (0.002)	0.084 (0.001)	0.104 (0.001)
	AIC	0.196 (0.004)	0.140 (0.002)	0.090 (0.002)	0.210 (0.004)	0.147 (0.002)	0.102 (0.002)	0.142 (0.001)
	BIC	0.187 (0.004)	0.135 (0.002)	0.093 (0.001)	0.196 (0.004)	0.141 (0.002)	0.104 (0.002)	0.139 (0.001)
	Oracle (Bayes)	0.151 (0.003)	0.084 (0.002)	0.040 (0.001)	0.157 (0.003)	0.087 (0.002)	0.039 (0.001)	0.101 (0.001)
	Oracle (freq)	0.167 (0.005)	0.080 (0.002)	0.039 (0.001)	0.184 (0.005)	0.087 (0.002)	0.039 (0.001)	0.106 (0.001)
Log-L	BMA	0.129 (0.004)	0.072 (0.002)	0.098 (0.001)	0.141 (0.003)	0.080 (0.002)	0.094 (0.001)	0.100 (0.001)
	BF	0.182 (0.004)	0.112 (0.002)	0.114 (0.001)	0.191 (0.003)	0.115 (0.002)	0.113 (0.001)	0.135 (0.001)
	AIC	0.157 (0.005)	0.114 (0.002)	0.124 (0.001)	0.183 (0.006)	0.125 (0.002)	0.125 (0.001)	0.132 (0.001)
	BIC	0.158 (0.005)	0.087 (0.002)	0.114 (0.001)	0.185 (0.006)	0.103 (0.002)	0.113 (0.001)	0.123 (0.001)
	Oracle (Bayes)	0.169 (0.004)	0.091 (0.002)	0.035 (0.001)	0.178 (0.003)	0.092 (0.002)	0.038 (0.001)	0.107 (0.001)
	Oracle (freq)	0.157 (0.005)	0.080 (0.002)	0.034 (0.001)	0.179 (0.006)	0.083 (0.002)	0.036 (0.001)	0.100 (0.002)
Gamma	BMA	0.234 (0.004)	0.145 (0.002)	0.085 (0.002)	0.239 (0.004)	0.156 (0.002)	0.098 (0.002)	0.160 (0.001)
	BF	0.269 (0.004)	0.171 (0.003)	0.101 (0.002)	0.271 (0.004)	0.173 (0.003)	0.110 (0.002)	0.184 (0.001)
	AIC	0.198 (0.008)	0.126 (0.003)	0.089 (0.002)	0.217 (0.008)	0.136 (0.003)	0.103 (0.002)	0.139 (0.002)
	BIC	0.204 (0.008)	0.108 (0.002)	0.088 (0.002)	0.222 (0.007)	0.126 (0.003)	0.100 (0.002)	0.134 (0.002)
	Oracle (Bayes)	0.204 (0.004)	0.089 (0.002)	0.031 (0.001)	0.211 (0.004)	0.102 (0.003)	0.041 (0.001)	0.123 (0.001)
	Oracle (freq)	0.173 (0.008)	0.064 (0.002)	0.028 (0.001)	0.192 (0.008)	0.082 (0.003)	0.038 (0.001)	0.102 (0.002)
Average	BMA	0.189 (0.002)	0.130 (0.001)	0.098 (0.001)	0.199 (0.002)	0.138 (0.001)	0.104 (0.001)	0.140 (0.000)
	BF	0.224 (0.002)	0.148 (0.001)	0.100 (0.001)	0.231 (0.002)	0.152 (0.001)	0.102 (0.001)	0.160 (0.001)
	AIC	0.197 (0.003)	0.137 (0.001)	0.100 (0.001)	0.215 (0.003)	0.144 (0.001)	0.105 (0.001)	0.145 (0.001)
	BIC	0.197 (0.003)	0.123 (0.001)	0.098 (0.001)	0.215 (0.003)	0.135 (0.001)	0.103 (0.001)	0.141 (0.001)
	Oracle (Bayes)	0.178 (0.002)	0.090 (0.001)	0.035 (0.000)	0.189 (0.002)	0.099 (0.001)	0.040 (0.001)	0.113 (0.001)
	Oracle (freq)	0.167 (0.003)	0.075 (0.001)	0.033 (0.000)	0.191 (0.003)	0.087 (0.001)	0.038 (0.000)	0.104 (0.001)

Table 15: Probability (SE) of finding support for the null hypothesis (H_0), alternative hypothesis (H_1), or not reaching a decision (undecided) in the simulated sequential analysis for each simulation condition comparing Bayesian model-averaging (BMA) to model selection using information (AIC, BIC), model selection using Bayes factors (BF), excluding the parametric models corresponding to the true data generating process, Bayesian or frequentist survival model with the true data generating survival function (Oracle), and Cox proportional hazard model. Different rows under AIC / BIC / Cox proportional hazard model correspond to the number of steps in the sequential analysis with binding asymmetric boundaries, Hwang-Shih-DeCani spending function, and $\alpha = 0.05$ for one-sided test.

		log(AF) = -.20			log(AF) = 0		
		H_0	Undecided	H_1	H_0	Undecided	H_1
BMA		0.998 (0.002)	0.000 (0.000)	0.002 (0.002)	0.896 (0.014)	0.048 (0.010)	0.056 (0.010)
BF		0.998 (0.002)	0.000 (0.000)	0.002 (0.002)	0.884 (0.014)	0.056 (0.010)	0.060 (0.011)
	2	1.000 (0.000)	0.000 (0.000)	0.000 (0.000)	0.594 (0.022)	0.388 (0.022)	0.018 (0.006)
	4	1.000 (0.000)	0.000 (0.000)	0.000 (0.000)	0.860 (0.016)	0.116 (0.014)	0.024 (0.007)
AIC	5	1.000 (0.000)	0.000 (0.000)	0.000 (0.000)	0.898 (0.014)	0.080 (0.012)	0.022 (0.007)
	10	1.000 (0.000)	0.000 (0.000)	0.000 (0.000)	0.924 (0.012)	0.044 (0.009)	0.032 (0.008)
	20	1.000 (0.000)	0.000 (0.000)	0.000 (0.000)	0.930 (0.011)	0.042 (0.009)	0.028 (0.007)
	2	1.000 (0.000)	0.000 (0.000)	0.000 (0.000)	0.592 (0.022)	0.390 (0.022)	0.018 (0.006)
	4	1.000 (0.000)	0.000 (0.000)	0.000 (0.000)	0.856 (0.016)	0.118 (0.014)	0.026 (0.007)
BIC	5	1.000 (0.000)	0.000 (0.000)	0.000 (0.000)	0.898 (0.014)	0.080 (0.012)	0.022 (0.007)
	10	1.000 (0.000)	0.000 (0.000)	0.000 (0.000)	0.926 (0.012)	0.042 (0.009)	0.032 (0.008)
	20	1.000 (0.000)	0.000 (0.000)	0.000 (0.000)	0.930 (0.011)	0.042 (0.009)	0.028 (0.007)
	2	1.000 (0.000)	0.000 (0.000)	0.000 (0.000)	0.592 (0.022)	0.390 (0.022)	0.018 (0.006)
	4	1.000 (0.000)	0.000 (0.000)	0.000 (0.000)	0.852 (0.016)	0.122 (0.015)	0.026 (0.007)
Cox	5	1.000 (0.000)	0.000 (0.000)	0.000 (0.000)	0.896 (0.014)	0.082 (0.012)	0.022 (0.007)
	10	1.000 (0.000)	0.000 (0.000)	0.000 (0.000)	0.930 (0.011)	0.036 (0.008)	0.034 (0.008)
	20	1.000 (0.000)	0.000 (0.000)	0.000 (0.000)	0.932 (0.011)	0.036 (0.008)	0.032 (0.008)
Oracle (Bayes)		0.998 (0.002)	0.000 (0.000)	0.002 (0.002)	0.900 (0.013)	0.040 (0.009)	0.060 (0.011)
Oracle (freq)	2	1.000 (0.000)	0.000 (0.000)	0.000 (0.000)	0.590 (0.022)	0.392 (0.022)	0.018 (0.006)
	4	1.000 (0.000)	0.000 (0.000)	0.000 (0.000)	0.852 (0.016)	0.124 (0.015)	0.024 (0.007)
	5	1.000 (0.000)	0.000 (0.000)	0.000 (0.000)	0.896 (0.014)	0.084 (0.012)	0.020 (0.006)
	10	1.000 (0.000)	0.000 (0.000)	0.000 (0.000)	0.928 (0.012)	0.038 (0.009)	0.034 (0.008)
	20	1.000 (0.000)	0.000 (0.000)	0.000 (0.000)	0.934 (0.011)	0.036 (0.008)	0.030 (0.008)

		log(AF) = 0.20			log(AF) = 0.40		
		H_0	Undecided	H_1	H_0	Undecided	H_1
BMA		0.184 (0.017)	0.136 (0.015)	0.680 (0.021)	0.016 (0.006)	0.008 (0.004)	0.976 (0.007)
BF		0.164 (0.017)	0.152 (0.016)	0.684 (0.021)	0.016 (0.006)	0.008 (0.004)	0.976 (0.007)
	2	0.038 (0.009)	0.706 (0.020)	0.256 (0.020)	0.002 (0.002)	0.088 (0.013)	0.910 (0.013)
	4	0.084 (0.012)	0.330 (0.021)	0.586 (0.022)	0.004 (0.003)	0.020 (0.006)	0.976 (0.007)
AIC	5	0.100 (0.013)	0.268 (0.020)	0.632 (0.022)	0.002 (0.002)	0.022 (0.007)	0.976 (0.007)
	10	0.114 (0.014)	0.234 (0.019)	0.652 (0.021)	0.004 (0.003)	0.012 (0.005)	0.984 (0.006)
	20	0.112 (0.014)	0.212 (0.018)	0.676 (0.021)	0.006 (0.003)	0.012 (0.005)	0.982 (0.006)
	2	0.038 (0.009)	0.706 (0.020)	0.256 (0.020)	0.002 (0.002)	0.084 (0.012)	0.914 (0.013)
	4	0.082 (0.012)	0.330 (0.021)	0.588 (0.022)	0.004 (0.003)	0.020 (0.006)	0.976 (0.007)
BIC	5	0.102 (0.014)	0.266 (0.020)	0.632 (0.022)	0.002 (0.002)	0.022 (0.007)	0.976 (0.007)
	10	0.116 (0.014)	0.232 (0.019)	0.652 (0.021)	0.004 (0.003)	0.012 (0.005)	0.984 (0.006)
	20	0.116 (0.014)	0.208 (0.018)	0.676 (0.021)	0.006 (0.003)	0.012 (0.005)	0.982 (0.006)
	2	0.038 (0.009)	0.692 (0.021)	0.270 (0.020)	0.002 (0.002)	0.082 (0.012)	0.916 (0.012)
	4	0.080 (0.012)	0.322 (0.021)	0.598 (0.022)	0.004 (0.003)	0.020 (0.006)	0.976 (0.007)
Cox	5	0.104 (0.014)	0.250 (0.019)	0.646 (0.021)	0.002 (0.002)	0.020 (0.006)	0.978 (0.007)
	10	0.116 (0.014)	0.222 (0.019)	0.662 (0.021)	0.004 (0.003)	0.010 (0.004)	0.986 (0.005)
	20	0.114 (0.014)	0.202 (0.018)	0.684 (0.021)	0.006 (0.003)	0.010 (0.004)	0.984 (0.006)
Oracle (Bayes)		0.190 (0.018)	0.130 (0.015)	0.680 (0.021)	0.018 (0.006)	0.002 (0.002)	0.980 (0.006)
Oracle (freq)	2	0.038 (0.009)	0.706 (0.020)	0.256 (0.020)	0.002 (0.002)	0.092 (0.013)	0.906 (0.013)
	4	0.082 (0.012)	0.332 (0.021)	0.586 (0.022)	0.004 (0.003)	0.018 (0.006)	0.978 (0.007)
	5	0.096 (0.013)	0.264 (0.020)	0.640 (0.021)	0.002 (0.002)	0.020 (0.006)	0.978 (0.007)
	10	0.118 (0.014)	0.228 (0.019)	0.654 (0.021)	0.004 (0.003)	0.010 (0.004)	0.986 (0.005)
	20	0.114 (0.014)	0.206 (0.018)	0.680 (0.021)	0.004 (0.003)	0.010 (0.004)	0.986 (0.005)

Table 16: Mean time in months (SE) of finding support for either the null or alternative hypothesis for each simulation condition comparing Bayesian model-averaging (BMA) to model selection using information (AIC, BIC), model selection using Bayes factors (BF), excluding the parametric models corresponding to the true data generating process, Bayesian or frequentist survival model with the true data generating survival function (Oracle), and Cox proportional hazard model. Different rows under AIC / BIC / Cox proportional hazard model correspond to the number of steps in the sequential analysis with binding asymmetric boundaries, Hwang-Shih-DeCani spending function, and $\alpha = 0.05$ for one-sided test.

**Time**		log(AF) = -0.20		log(AF) = 0		Average		
		H_0	H_1	H_0	H_0	H_0	H_1	H_0 or H_1
BMA		5.3 (0.14)	4.0 (–)	10.6 (0.36)	7.0 (0.96)	7.8 (0.21)	6.9 (0.93)	7.8 (0.20)
BF		5.6 (0.15)	4.0 (–)	11.4 (0.41)	8.1 (0.97)	8.3 (0.23)	7.9 (0.95)	8.3 (0.22)
	2	16.9 (0.27)	– (–)	20.3 (0.55)	31.9 (7.04)	18.1 (0.27)	31.9 (7.04)	18.3 (0.28)
	4	10.8 (0.19)	– (–)	21.1 (0.46)	26.2 (5.16)	15.6 (0.29)	26.2 (5.16)	15.7 (0.30)
AIC	5	10.3 (0.20)	– (–)	20.8 (0.48)	33.2 (5.54)	15.3 (0.30)	33.2 (5.54)	15.5 (0.31)
	10	9.0 (0.18)	– (–)	20.1 (0.52)	36.2 (4.68)	14.3 (0.32)	36.2 (4.68)	14.7 (0.34)
	20	8.5 (0.18)	– (–)	19.6 (0.52)	29.3 (4.60)	13.9 (0.32)	29.3 (4.60)	14.1 (0.33)
	2	16.8 (0.25)	– (–)	20.2 (0.53)	31.9 (7.04)	18.0 (0.26)	31.9 (7.04)	18.2 (0.27)
	4	10.8 (0.19)	– (–)	20.9 (0.46)	25.0 (4.87)	15.5 (0.29)	25.0 (4.87)	15.6 (0.29)
BIC	5	10.2 (0.20)	– (–)	20.8 (0.47)	33.2 (5.54)	15.2 (0.30)	33.2 (5.54)	15.4 (0.31)
	10	9.0 (0.18)	– (–)	20.0 (0.51)	36.3 (4.65)	14.2 (0.32)	36.3 (4.65)	14.6 (0.33)
	20	8.4 (0.18)	– (–)	19.6 (0.52)	29.0 (4.57)	13.8 (0.32)	29.0 (4.57)	14.0 (0.33)
	2	16.8 (0.25)	– (–)	20.3 (0.55)	31.9 (7.04)	18.1 (0.26)	31.9 (7.04)	18.2 (0.28)
	4	10.8 (0.19)	– (–)	20.9 (0.46)	25.0 (4.87)	15.5 (0.29)	25.0 (4.87)	15.6 (0.29)
Cox	5	10.2 (0.20)	– (–)	20.7 (0.48)	33.2 (5.54)	15.2 (0.30)	33.2 (5.54)	15.4 (0.31)
	10	8.9 (0.18)	– (–)	20.0 (0.51)	36.9 (4.53)	14.3 (0.32)	36.9 (4.53)	14.6 (0.34)
	20	8.4 (0.18)	– (–)	19.5 (0.51)	29.9 (4.34)	13.7 (0.32)	29.9 (4.34)	14.0 (0.32)
Oracle (Bayes)		5.3 (0.13)	4.0 (–)	10.4 (0.33)	7.0 (0.86)	7.8 (0.19)	6.9 (0.84)	7.7 (0.19)
	2	16.8 (0.25)	– (–)	20.4 (0.55)	31.9 (7.04)	18.1 (0.27)	31.9 (7.04)	18.2 (0.28)
	4	10.8 (0.19)	– (–)	20.9 (0.46)	26.8 (5.01)	15.4 (0.29)	26.8 (5.01)	15.6 (0.29)
Oracle (freq)	5	10.2 (0.20)	– (–)	20.7 (0.48)	34.4 (5.99)	15.2 (0.30)	34.4 (5.99)	15.4 (0.31)
	10	8.9 (0.18)	– (–)	20.0 (0.51)	37.3 (4.40)	14.2 (0.32)	37.3 (4.40)	14.6 (0.34)
	20	8.5 (0.17)	– (–)	19.6 (0.51)	30.6 (4.17)	13.9 (0.32)	30.6 (4.17)	14.1 (0.32)

**Time**		log(AF) = 0.20		log(AF) = 0.40		Average		
		H_0	H_1	H_0	H_0	H_0	H_1	H_0 or H_1
BMA		11.1 (0.76)	14.0 (0.55)	7.8 (1.31)	8.7 (0.27)	10.8 (0.71)	10.8 (0.29)	10.8 (0.27)
BF		11.6 (0.89)	13.9 (0.54)	8.8 (1.31)	8.7 (0.26)	11.4 (0.82)	10.8 (0.28)	10.9 (0.27)
	2	20.3 (0.31)	20.4 (0.12)	24.4 (–)	22.7 (0.07)	20.5 (0.36)	22.2 (0.07)	22.1 (0.07)
	4	26.7 (1.78)	30.0 (0.66)	17.8 (6.66)	21.6 (0.49)	26.3 (1.73)	24.7 (0.42)	24.8 (0.41)
AIC	5	30.1 (2.21)	32.7 (0.84)	30.1 (–)	19.8 (0.40)	30.1 (2.17)	24.9 (0.47)	25.2 (0.46)
	10	29.3 (2.24)	29.2 (0.73)	14.5 (9.96)	17.7 (0.34)	28.8 (2.21)	22.3 (0.41)	22.8 (0.41)
	20	29.7 (2.15)	28.5 (0.71)	12.5 (5.07)	16.9 (0.32)	28.8 (2.11)	21.6 (0.40)	22.1 (0.40)
	2	20.3 (0.31)	20.4 (0.12)	24.4 (–)	22.7 (0.07)	20.5 (0.36)	22.2 (0.07)	22.1 (0.07)
	4	27.7 (1.93)	29.9 (0.66)	17.8 (6.66)	21.3 (0.48)	27.2 (1.88)	24.5 (0.42)	24.7 (0.41)
BIC	5	29.8 (2.20)	32.3 (0.85)	30.1 (–)	19.4 (0.41)	29.8 (2.16)	24.5 (0.47)	24.8 (0.46)
	10	28.8 (2.23)	29.2 (0.74)	14.5 (9.96)	17.6 (0.34)	28.4 (2.20)	22.2 (0.41)	22.6 (0.41)
	20	28.9 (2.08)	28.3 (0.72)	11.9 (5.57)	16.6 (0.33)	28.1 (2.05)	21.4 (0.40)	21.9 (0.41)
	2	20.3 (0.31)	20.4 (0.11)	24.4 (–)	22.7 (0.07)	20.5 (0.36)	22.2 (0.07)	22.1 (0.07)
	4	27.5 (1.97)	30.0 (0.67)	17.8 (6.66)	21.2 (0.48)	27.0 (1.92)	24.6 (0.42)	24.7 (0.41)
Cox	5	30.3 (2.20)	32.2 (0.84)	30.1 (–)	19.3 (0.42)	30.3 (2.16)	24.4 (0.47)	24.8 (0.47)
	10	28.5 (2.18)	29.1 (0.74)	14.5 (9.96)	17.5 (0.35)	28.0 (2.15)	22.2 (0.41)	22.6 (0.41)
	20	28.7 (2.07)	28.2 (0.72)	11.9 (5.57)	16.5 (0.33)	27.8 (2.04)	21.3 (0.41)	21.8 (0.41)
Oracle (Bayes)		10.7 (0.68)	13.7 (0.53)	8.2 (1.14)	8.6 (0.25)	10.5 (0.63)	10.7 (0.28)	10.7 (0.26)
	2	20.3 (0.31)	20.4 (0.12)	24.4 (–)	22.7 (0.07)	20.5 (0.36)	22.2 (0.07)	22.1 (0.07)
	4	27.7 (1.93)	30.3 (0.67)	17.8 (6.66)	21.7 (0.49)	27.2 (1.88)	24.9 (0.42)	25.1 (0.41)
Oracle (freq)	5	30.1 (2.27)	32.7 (0.82)	30.1 (–)	20.1 (0.40)	30.1 (2.22)	25.1 (0.46)	25.4 (0.45)
	10	28.7 (2.17)	29.6 (0.72)	14.5 (9.96)	18.2 (0.34)	28.2 (2.14)	22.7 (0.40)	23.1 (0.40)
	20	29.4 (2.08)	28.8 (0.70)	16.5 (5.40)	17.2 (0.33)	29.0 (2.03)	21.9 (0.40)	22.4 (0.40)

2 Detailed Simulation Results Including the True Parametric Family

List of the included tables:

- Table 17 and 18: Bias of the log(AF) estimate (fixed-n).
- Table 19 and 20: RMSE of the log(AF) estimate (fixed-n).
- Table 21 and 22: Error rate and power when making decisions about the presence of the treatment effect (fixed-n).
- Table 23 and 24: Confidence interval coverage of the log(AF) estimate (fixed-n).
- Table 25 and 26: Bias of predicted survival at 20 years effect across the simulation settings (fixed-n).
- Table 27 and 28: RMSE of predicted survival at 20 years effect across the simulation settings (fixed-n).
- Table 29: Error rate and power when making decisions about the presence of the treatment effect (sequential-n).
- Table 30: Time to decide for the sequential design across the simulation settings.

Table 17: Bias (SE) for each simulation condition (Part 1) comparing Bayesian model-averaging (BMA) to model selection using information (AIC, BIC), and model selection using Bayes factors (BF), including the parametric models corresponding to the true data generating process. Rows correspond to different simulation conditions according to the true parametric family (Exp = exponential, Weib = Weibull, Log-N = log-normal, Log-L = Log-logistic, Gamma = gamma). Average corresponds to the results aggregated across distributions (last row), log(AF) $\times$ N (last column), or the whole simulation (bottom right corner).

**Bias**		log(AF) = -0.20			log(AF) = 0		
		N = 50	N = 200	N = 1000	N = 50	N = 200	N = 1000
Exp	BMA	0.039 (0.021)	-0.031 (0.012)	0.008 (0.006)	0.017 (0.021)	0.019 (0.013)	-0.004 (0.006)
	BF	0.043 (0.021)	-0.025 (0.011)	0.010 (0.006)	0.026 (0.021)	0.019 (0.013)	-0.003 (0.006)
	AIC	-0.015 (0.034)	-0.036 (0.012)	0.008 (0.006)	0.009 (0.063)	0.024 (0.014)	-0.002 (0.007)
	BIC	-0.011 (0.033)	-0.032 (0.012)	0.010 (0.006)	0.012 (0.066)	0.025 (0.014)	-0.002 (0.006)
Weib	BMA	-0.022 (0.019)	0.003 (0.012)	-0.006 (0.005)	-0.005 (0.021)	0.000 (0.012)	-0.003 (0.006)
	BF	-0.018 (0.019)	0.006 (0.012)	-0.004 (0.005)	-0.003 (0.020)	0.002 (0.012)	-0.003 (0.006)
	AIC	-0.073 (0.025)	0.006 (0.012)	-0.001 (0.005)	-0.005 (0.028)	0.005 (0.012)	-0.002 (0.005)
	BIC	-0.084 (0.026)	-0.003 (0.013)	-0.007 (0.005)	0.002 (0.028)	0.005 (0.013)	-0.002 (0.006)
Log-N	BMA	0.018 (0.021)	0.004 (0.012)	0.009 (0.005)	0.003 (0.020)	0.017 (0.013)	0.001 (0.005)
	BF	0.021 (0.021)	0.004 (0.012)	0.008 (0.005)	0.004 (0.020)	0.017 (0.013)	0.001 (0.005)
	AIC	-0.046 (0.079)	0.002 (0.012)	0.009 (0.005)	0.103 (0.064)	0.022 (0.013)	0.002 (0.005)
	BIC	-0.037 (0.088)	0.000 (0.013)	0.009 (0.005)	0.093 (0.065)	0.022 (0.014)	0.002 (0.005)
Log-L	BMA	-0.009 (0.021)	-0.014 (0.012)	-0.011 (0.005)	-0.009 (0.021)	0.013 (0.012)	0.002 (0.005)
	BF	-0.007 (0.020)	-0.012 (0.012)	-0.010 (0.005)	-0.006 (0.021)	0.016 (0.012)	0.002 (0.005)
	AIC	-0.075 (0.039)	-0.013 (0.013)	-0.009 (0.005)	0.040 (0.051)	0.020 (0.013)	0.003 (0.005)
	BIC	-0.104 (0.051)	-0.020 (0.013)	-0.011 (0.005)	0.009 (0.066)	0.020 (0.013)	0.003 (0.005)
Gamma	BMA	0.011 (0.019)	-0.004 (0.012)	-0.002 (0.005)	0.007 (0.020)	-0.015 (0.012)	0.006 (0.005)
	BF	0.019 (0.019)	0.001 (0.012)	-0.001 (0.005)	0.006 (0.020)	-0.013 (0.012)	0.007 (0.005)
	AIC	-0.018 (0.026)	-0.001 (0.012)	0.002 (0.005)	0.009 (0.029)	-0.009 (0.012)	0.007 (0.005)
	BIC	-0.035 (0.026)	-0.012 (0.013)	-0.001 (0.005)	0.018 (0.030)	-0.008 (0.013)	0.008 (0.006)
Average	BMA	0.007 (0.009)	-0.008 (0.005)	0.000 (0.002)	0.003 (0.009)	0.007 (0.006)	0.000 (0.003)
	BF	0.012 (0.009)	-0.005 (0.005)	0.000 (0.002)	0.005 (0.009)	0.008 (0.006)	0.001 (0.003)
	AIC	-0.045 (0.020)	-0.008 (0.006)	0.002 (0.002)	0.031 (0.022)	0.013 (0.006)	0.002 (0.003)
	BIC	-0.054 (0.023)	-0.014 (0.006)	0.000 (0.002)	0.027 (0.024)	0.013 (0.006)	0.002 (0.003)

Table 18: Bias (SE) for each simulation condition (Part 2) comparing Bayesian model-averaging (BMA) to model selection using information (AIC, BIC), and model selection using Bayes factors (BF), including the parametric models corresponding to the true data generating process. Rows correspond to different simulation conditions according to the true parametric family (Exp = exponential, Weib = Weibull, Log-N = log-normal, Log-L = Log-logistic, Gamma = gamma). Average corresponds to the results aggregated across distributions (last row), log(AF) × N (last column), or the whole simulation (bottom right corner).

**Bias**		log(AF) = 0.20			log(AF) = 0.40			Average
		N = 50	N = 200	N = 1000	N = 50	N = 200	N = 1000	
Exp	BMA	-0.068 (0.023)	-0.007 (0.014)	-0.011 (0.006)	-0.098 (0.022)	-0.040 (0.015)	-0.013 (0.007)	-0.016 (0.004)
	BF	-0.063 (0.023)	-0.008 (0.014)	-0.012 (0.006)	-0.091 (0.022)	-0.045 (0.015)	-0.017 (0.007)	-0.014 (0.004)
	AIC	0.139 (0.107)	0.012 (0.015)	-0.008 (0.007)	0.291 (0.120)	-0.007 (0.017)	-0.007 (0.007)	0.034 (0.015)
	BIC	0.148 (0.106)	0.006 (0.015)	-0.010 (0.006)	0.335 (0.132)	-0.014 (0.016)	-0.013 (0.007)	0.038 (0.016)
Weib	BMA	-0.059 (0.019)	0.006 (0.012)	0.007 (0.006)	-0.099 (0.022)	0.026 (0.013)	0.015 (0.006)	-0.011 (0.004)
	BF	-0.055 (0.019)	0.006 (0.012)	0.007 (0.006)	-0.097 (0.021)	0.023 (0.013)	0.014 (0.006)	-0.010 (0.004)
	AIC	0.099 (0.076)	0.018 (0.013)	0.005 (0.006)	0.252 (0.105)	0.046 (0.014)	0.008 (0.006)	0.030 (0.011)
	BIC	0.100 (0.076)	0.027 (0.013)	0.014 (0.006)	0.337 (0.119)	0.070 (0.015)	0.028 (0.006)	0.040 (0.013)
Log-N	BMA	-0.040 (0.021)	-0.006 (0.012)	-0.007 (0.005)	-0.104 (0.021)	-0.040 (0.013)	-0.006 (0.006)	-0.013 (0.004)
	BF	-0.041 (0.021)	-0.003 (0.012)	-0.004 (0.005)	-0.105 (0.021)	-0.036 (0.013)	-0.002 (0.006)	-0.011 (0.004)
	AIC	-0.005 (0.089)	0.006 (0.012)	-0.003 (0.005)	0.209 (0.109)	-0.021 (0.014)	-0.002 (0.006)	0.023 (0.015)
	BIC	-0.018 (0.098)	0.015 (0.013)	-0.003 (0.005)	0.322 (0.129)	-0.009 (0.014)	-0.002 (0.006)	0.033 (0.016)
Log-L	BMA	-0.035 (0.020)	-0.014 (0.012)	0.008 (0.006)	-0.064 (0.021)	-0.003 (0.013)	-0.008 (0.006)	-0.012 (0.004)
	BF	-0.030 (0.020)	-0.011 (0.012)	0.010 (0.006)	-0.068 (0.021)	-0.005 (0.013)	-0.006 (0.006)	-0.011 (0.004)
	AIC	0.143 (0.076)	0.001 (0.013)	0.009 (0.006)	0.486 (0.131)	0.009 (0.014)	-0.011 (0.006)	0.050 (0.014)
	BIC	0.147 (0.076)	0.003 (0.013)	0.013 (0.006)	0.582 (0.143)	0.034 (0.014)	-0.002 (0.006)	0.056 (0.016)
Gamma	BMA	-0.020 (0.021)	0.004 (0.012)	0.016 (0.006)	-0.064 (0.021)	0.021 (0.013)	0.018 (0.006)	-0.002 (0.004)
	BF	-0.015 (0.021)	0.004 (0.012)	0.015 (0.006)	-0.066 (0.021)	0.018 (0.013)	0.016 (0.006)	-0.001 (0.004)
	AIC	0.261 (0.106)	0.013 (0.013)	0.012 (0.006)	0.463 (0.132)	0.037 (0.014)	0.011 (0.006)	0.066 (0.015)
	BIC	0.253 (0.112)	0.025 (0.014)	0.018 (0.006)	0.480 (0.132)	0.065 (0.014)	0.025 (0.006)	0.070 (0.015)
Average	BMA	-0.044 (0.009)	-0.003 (0.006)	0.003 (0.003)	-0.086 (0.010)	-0.007 (0.006)	0.001 (0.003)	-0.011 (0.002)
	BF	-0.041 (0.009)	-0.002 (0.006)	0.003 (0.003)	-0.085 (0.010)	-0.009 (0.006)	0.001 (0.003)	-0.009 (0.002)
	AIC	0.127 (0.041)	0.010 (0.006)	0.003 (0.003)	0.340 (0.054)	0.013 (0.006)	0.000 (0.003)	0.041 (0.006)
	BIC	0.126 (0.042)	0.015 (0.006)	0.006 (0.003)	0.411 (0.059)	0.029 (0.007)	0.007 (0.003)	0.047 (0.007)

Table 19: RMSE (SE) for each simulation condition (Part 1) comparing Bayesian model-averaging (BMA) to model selection using information (AIC, BIC), and model selection using Bayes factors (BF), including the parametric models corresponding to the true data generating process. Rows correspond to different simulation conditions according to the true parametric family (Exp = exponential, Weib = Weibull, Log-N = log-normal, Log-L = Log-logistic, Gamma = gamma). Average corresponds to the results aggregated across distributions (last row), log(AF) $\times$ N (last column), or the whole simulation (bottom right corner).

**RMSE**		log(AF) = -0.20			log(AF) = 0		
		N = 50	N = 200	N = 1000	N = 50	N = 200	N = 1000
Exp	BMA	0.466 (0.013)	0.261 (0.009)	0.136 (0.004)	0.469 (0.014)	0.301 (0.009)	0.145 (0.004)
	BF	0.464 (0.013)	0.257 (0.009)	0.136 (0.004)	0.468 (0.014)	0.297 (0.009)	0.144 (0.004)
	AIC	0.749 (0.052)	0.278 (0.010)	0.137 (0.004)	1.417 (0.418)	0.323 (0.010)	0.147 (0.004)
	BIC	0.731 (0.053)	0.269 (0.009)	0.137 (0.004)	1.472 (0.445)	0.312 (0.010)	0.145 (0.004)
Weib	BMA	0.421 (0.013)	0.265 (0.010)	0.114 (0.004)	0.459 (0.014)	0.268 (0.009)	0.124 (0.004)
	BF	0.418 (0.013)	0.264 (0.010)	0.113 (0.004)	0.455 (0.014)	0.269 (0.009)	0.123 (0.004)
	AIC	0.555 (0.019)	0.275 (0.012)	0.112 (0.004)	0.629 (0.024)	0.278 (0.010)	0.122 (0.004)
	BIC	0.587 (0.023)	0.287 (0.011)	0.115 (0.004)	0.635 (0.023)	0.288 (0.010)	0.127 (0.004)
Log-N	BMA	0.464 (0.017)	0.259 (0.008)	0.116 (0.004)	0.454 (0.014)	0.282 (0.009)	0.121 (0.004)
	BF	0.458 (0.018)	0.260 (0.008)	0.116 (0.004)	0.449 (0.015)	0.283 (0.009)	0.122 (0.004)
	AIC	1.775 (0.459)	0.271 (0.008)	0.117 (0.004)	1.437 (0.471)	0.294 (0.010)	0.122 (0.004)
	BIC	1.976 (0.471)	0.280 (0.008)	0.117 (0.004)	1.444 (0.468)	0.304 (0.010)	0.122 (0.004)
Log-L	BMA	0.461 (0.016)	0.272 (0.010)	0.115 (0.004)	0.464 (0.015)	0.273 (0.009)	0.115 (0.004)
	BF	0.457 (0.016)	0.274 (0.010)	0.115 (0.004)	0.460 (0.015)	0.275 (0.009)	0.115 (0.004)
	AIC	0.872 (0.220)	0.283 (0.010)	0.115 (0.004)	1.147 (0.450)	0.285 (0.010)	0.114 (0.004)
	BIC	1.148 (0.476)	0.294 (0.011)	0.116 (0.004)	1.467 (0.457)	0.295 (0.010)	0.116 (0.004)
Gamma	BMA	0.428 (0.014)	0.261 (0.008)	0.110 (0.004)	0.457 (0.014)	0.268 (0.009)	0.121 (0.004)
	BF	0.426 (0.014)	0.262 (0.008)	0.110 (0.004)	0.455 (0.014)	0.270 (0.009)	0.121 (0.004)
	AIC	0.581 (0.026)	0.272 (0.009)	0.109 (0.004)	0.649 (0.027)	0.278 (0.010)	0.120 (0.004)
	BIC	0.591 (0.023)	0.280 (0.009)	0.112 (0.004)	0.670 (0.027)	0.288 (0.010)	0.123 (0.004)
Average	BMA	0.448 (0.007)	0.264 (0.004)	0.118 (0.002)	0.460 (0.006)	0.279 (0.004)	0.126 (0.002)
	BF	0.445 (0.007)	0.263 (0.004)	0.118 (0.002)	0.457 (0.006)	0.279 (0.004)	0.125 (0.002)
	AIC	1.012 (0.160)	0.276 (0.004)	0.118 (0.002)	1.114 (0.167)	0.292 (0.004)	0.126 (0.002)
	BIC	1.136 (0.179)	0.282 (0.004)	0.120 (0.002)	1.204 (0.176)	0.297 (0.005)	0.127 (0.002)

Table 20: RMSE (SE) for each simulation condition (Part 2) comparing Bayesian model-averaging (BMA) to model selection using information (AIC, BIC), and model selection using Bayes factors (BF), including the parametric models corresponding to the true data generating process. Rows correspond to different simulation conditions according to the true parametric family (Exp = exponential, Weib = Weibull, Log-N = log-normal, Log-L = Log-logistic, Gamma = gamma). Average corresponds to the results aggregated across distributions (last row), log(AF) × N (last column), or the whole simulation (bottom right corner).

**RMSE**		log(AF) = 0.20			log(AF) = 0.40			Average
		N = 50	N = 200	N = 1000	N = 50	N = 200	N = 1000	
Exp	BMA	0.511 (0.016)	0.311 (0.011)	0.144 (0.005)	0.507 (0.015)	0.331 (0.011)	0.152 (0.005)	0.342 (0.004)
	BF	0.507 (0.016)	0.307 (0.011)	0.144 (0.005)	0.507 (0.015)	0.330 (0.011)	0.151 (0.005)	0.340 (0.004)
	AIC	2.394 (0.435)	0.346 (0.013)	0.147 (0.005)	2.689 (0.427)	0.371 (0.014)	0.156 (0.005)	1.157 (0.117)
	BIC	2.378 (0.438)	0.326 (0.012)	0.143 (0.005)	2.971 (0.444)	0.350 (0.012)	0.151 (0.005)	1.213 (0.121)
Weib	BMA	0.437 (0.014)	0.273 (0.009)	0.129 (0.004)	0.493 (0.015)	0.293 (0.010)	0.126 (0.005)	0.314 (0.004)
	BF	0.433 (0.014)	0.274 (0.009)	0.130 (0.004)	0.488 (0.014)	0.294 (0.010)	0.126 (0.005)	0.312 (0.004)
	AIC	1.697 (0.462)	0.293 (0.011)	0.128 (0.004)	2.350 (0.446)	0.322 (0.013)	0.124 (0.005)	0.890 (0.119)
	BIC	1.703 (0.460)	0.295 (0.010)	0.136 (0.004)	2.684 (0.451)	0.333 (0.012)	0.135 (0.005)	0.970 (0.122)
Log-N	BMA	0.469 (0.013)	0.266 (0.008)	0.117 (0.003)	0.482 (0.015)	0.290 (0.009)	0.134 (0.004)	0.321 (0.004)
	BF	0.464 (0.013)	0.265 (0.008)	0.118 (0.004)	0.479 (0.015)	0.290 (0.009)	0.134 (0.004)	0.319 (0.004)
	AIC	1.991 (0.451)	0.276 (0.009)	0.118 (0.004)	2.443 (0.431)	0.307 (0.010)	0.135 (0.004)	1.138 (0.122)
	BIC	2.181 (0.460)	0.294 (0.010)	0.118 (0.004)	2.895 (0.440)	0.319 (0.011)	0.135 (0.004)	1.276 (0.126)
Log-L	BMA	0.456 (0.016)	0.274 (0.008)	0.125 (0.004)	0.473 (0.015)	0.291 (0.009)	0.132 (0.004)	0.320 (0.004)
	BF	0.451 (0.016)	0.275 (0.008)	0.127 (0.004)	0.467 (0.015)	0.293 (0.010)	0.132 (0.004)	0.318 (0.004)
	AIC	1.693 (0.454)	0.289 (0.009)	0.126 (0.004)	2.959 (0.428)	0.308 (0.011)	0.132 (0.004)	1.084 (0.118)
	BIC	1.703 (0.451)	0.296 (0.009)	0.130 (0.004)	3.253 (0.437)	0.323 (0.011)	0.136 (0.004)	1.204 (0.122)
Gamma	BMA	0.472 (0.017)	0.278 (0.009)	0.127 (0.004)	0.480 (0.016)	0.288 (0.009)	0.128 (0.004)	0.317 (0.004)
	BF	0.472 (0.017)	0.277 (0.009)	0.127 (0.004)	0.476 (0.016)	0.287 (0.009)	0.127 (0.004)	0.316 (0.004)
	AIC	2.389 (0.440)	0.287 (0.010)	0.124 (0.004)	2.992 (0.444)	0.310 (0.011)	0.126 (0.004)	1.148 (0.122)
	BIC	2.516 (0.450)	0.303 (0.010)	0.130 (0.004)	2.998 (0.443)	0.327 (0.011)	0.137 (0.004)	1.174 (0.123)
Average	BMA	0.469 (0.007)	0.281 (0.004)	0.129 (0.002)	0.487 (0.007)	0.299 (0.005)	0.135 (0.002)	0.323 (0.002)
	BF	0.466 (0.007)	0.280 (0.004)	0.129 (0.002)	0.484 (0.007)	0.299 (0.004)	0.134 (0.002)	0.321 (0.002)
	AIC	2.056 (0.191)	0.299 (0.005)	0.129 (0.002)	2.699 (0.190)	0.324 (0.005)	0.135 (0.002)	1.088 (0.053)
	BIC	2.123 (0.194)	0.303 (0.005)	0.132 (0.002)	2.966 (0.194)	0.330 (0.005)	0.139 (0.002)	1.172 (0.054)

Table 21: Error rate / power (SE) for each simulation condition (Part 1) comparing Bayesian model-averaging (BMA) to model selection using information (AIC, BIC), and model selection using Bayes factors (BF), including the parametric models corresponding to the true data generating process. Rows correspond to different simulation conditions according to the true parametric family (Exp = exponential, Weib = Weibull, Log-N = log-normal, Log-L = Log-logistic, Gamma = gamma). Average corresponds to the results aggregated across distributions (last row), log(AF) × N (last column), or the whole simulation (bottom right corner).

**Error**		log(AF) = 0.20			log(AF) = 0.40			Average
		N = 50	N = 200	N = 1000	N = 50	N = 200	N = 1000	
Exp	BMA	0.022 (0.007)	0.008 (0.004)	0.000 (0.000)	0.066 (0.011)	0.098 (0.013)	0.058 (0.010)	0.074 (0.007)
	BF	0.024 (0.007)	0.004 (0.003)	0.000 (0.000)	0.062 (0.011)	0.094 (0.013)	0.058 (0.010)	0.071 (0.007)
	AIC	0.018 (0.006)	0.002 (0.002)	0.000 (0.000)	0.064 (0.011)	0.070 (0.011)	0.056 (0.010)	0.063 (0.006)
	BIC	0.016 (0.006)	0.002 (0.002)	0.000 (0.000)	0.058 (0.010)	0.068 (0.011)	0.054 (0.010)	0.060 (0.006)
Weib	BMA	0.016 (0.006)	0.016 (0.006)	0.000 (0.000)	0.068 (0.011)	0.076 (0.012)	0.056 (0.010)	0.067 (0.006)
	BF	0.018 (0.006)	0.016 (0.006)	0.000 (0.000)	0.066 (0.011)	0.070 (0.011)	0.060 (0.011)	0.065 (0.006)
	AIC	0.018 (0.006)	0.012 (0.005)	0.000 (0.000)	0.056 (0.010)	0.054 (0.010)	0.062 (0.011)	0.057 (0.006)
	BIC	0.016 (0.006)	0.014 (0.005)	0.000 (0.000)	0.052 (0.010)	0.048 (0.010)	0.060 (0.011)	0.053 (0.006)
Log-N	BMA	0.024 (0.007)	0.018 (0.006)	0.000 (0.000)	0.042 (0.009)	0.098 (0.013)	0.034 (0.008)	0.058 (0.006)
	BF	0.022 (0.007)	0.012 (0.005)	0.000 (0.000)	0.052 (0.010)	0.092 (0.013)	0.034 (0.008)	0.059 (0.006)
	AIC	0.022 (0.007)	0.008 (0.004)	0.000 (0.000)	0.040 (0.009)	0.072 (0.012)	0.032 (0.008)	0.048 (0.006)
	BIC	0.020 (0.006)	0.010 (0.004)	0.000 (0.000)	0.034 (0.008)	0.072 (0.012)	0.032 (0.008)	0.046 (0.005)
Log-L	BMA	0.024 (0.007)	0.014 (0.005)	0.000 (0.000)	0.054 (0.010)	0.084 (0.012)	0.048 (0.010)	0.062 (0.006)
	BF	0.028 (0.007)	0.010 (0.004)	0.000 (0.000)	0.056 (0.010)	0.092 (0.013)	0.046 (0.009)	0.065 (0.006)
	AIC	0.022 (0.007)	0.008 (0.004)	0.000 (0.000)	0.052 (0.010)	0.070 (0.011)	0.048 (0.010)	0.057 (0.006)
	BIC	0.022 (0.007)	0.008 (0.004)	0.000 (0.000)	0.046 (0.009)	0.062 (0.011)	0.048 (0.010)	0.052 (0.006)
Gamma	BMA	0.022 (0.007)	0.016 (0.006)	0.000 (0.000)	0.060 (0.011)	0.076 (0.012)	0.056 (0.010)	0.064 (0.006)
	BF	0.020 (0.006)	0.020 (0.006)	0.000 (0.000)	0.062 (0.011)	0.072 (0.012)	0.052 (0.010)	0.062 (0.006)
	AIC	0.020 (0.006)	0.012 (0.005)	0.000 (0.000)	0.050 (0.010)	0.052 (0.010)	0.056 (0.010)	0.053 (0.006)
	BIC	0.018 (0.006)	0.012 (0.005)	0.000 (0.000)	0.046 (0.009)	0.052 (0.010)	0.056 (0.010)	0.051 (0.006)
Average	BMA	0.022 (0.007)	0.016 (0.006)	0.000 (0.000)	0.060 (0.011)	0.076 (0.012)	0.056 (0.010)	0.065 (0.003)
	BF	0.022 (0.007)	0.016 (0.006)	0.000 (0.000)	0.060 (0.011)	0.076 (0.012)	0.056 (0.010)	0.065 (0.003)
	AIC	0.020 (0.003)	0.008 (0.002)	0.000 (0.000)	0.052 (0.004)	0.064 (0.005)	0.051 (0.004)	0.056 (0.003)
	BIC	0.018 (0.003)	0.009 (0.002)	0.000 (0.000)	0.047 (0.004)	0.060 (0.005)	0.050 (0.004)	0.053 (0.003)

Table 22: Error rate / power (SE) for each simulation condition (Part 2) comparing Bayesian model-averaging (BMA) to model selection using information (AIC, BIC), and model selection using Bayes factors (BF), including the parametric models corresponding to the true data generating process. Rows correspond to different simulation conditions according to the true parametric family (Exp = exponential, Weib = Weibull, Log-N = log-normal, Log-L = Log-logistic, Gamma = gamma). Average corresponds to the results aggregated across distributions (last row), log(AF) × N (last column), or the whole simulation (bottom right corner).

**Power**		log(AF) = 0.20			log(AF) = 0.40			Average
		N = 50	N = 200	N = 1000	N = 50	N = 200	N = 1000	
Exp	BMA	0.098 (0.013)	0.210 (0.018)	0.420 (0.022)	0.164 (0.017)	0.394 (0.022)	0.870 (0.015)	0.243 (0.006)
	BF	0.094 (0.013)	0.186 (0.017)	0.418 (0.022)	0.162 (0.016)	0.378 (0.022)	0.870 (0.015)	0.237 (0.006)
	AIC	0.082 (0.012)	0.136 (0.015)	0.370 (0.022)	0.136 (0.015)	0.326 (0.021)	0.842 (0.016)	0.212 (0.006)
	BIC	0.076 (0.012)	0.134 (0.015)	0.366 (0.022)	0.130 (0.015)	0.332 (0.021)	0.842 (0.016)	0.211 (0.006)
Weib	BMA	0.066 (0.011)	0.228 (0.019)	0.510 (0.022)	0.170 (0.017)	0.486 (0.022)	0.958 (0.009)	0.272 (0.007)
	BF	0.066 (0.011)	0.222 (0.019)	0.516 (0.022)	0.168 (0.017)	0.474 (0.022)	0.958 (0.009)	0.271 (0.007)
	AIC	0.062 (0.011)	0.186 (0.017)	0.522 (0.022)	0.144 (0.016)	0.402 (0.022)	0.948 (0.010)	0.255 (0.006)
	BIC	0.058 (0.010)	0.174 (0.017)	0.522 (0.022)	0.136 (0.015)	0.390 (0.022)	0.948 (0.010)	0.251 (0.006)
Log-N	BMA	0.102 (0.014)	0.206 (0.018)	0.450 (0.022)	0.144 (0.016)	0.434 (0.022)	0.920 (0.012)	0.255 (0.007)
	BF	0.096 (0.013)	0.196 (0.018)	0.456 (0.022)	0.146 (0.016)	0.410 (0.022)	0.922 (0.012)	0.251 (0.006)
	AIC	0.092 (0.013)	0.170 (0.017)	0.446 (0.022)	0.126 (0.015)	0.374 (0.022)	0.916 (0.012)	0.239 (0.006)
	BIC	0.082 (0.012)	0.170 (0.017)	0.446 (0.022)	0.102 (0.014)	0.350 (0.021)	0.916 (0.012)	0.233 (0.006)
Log-L	BMA	0.096 (0.013)	0.228 (0.019)	0.508 (0.022)	0.148 (0.016)	0.470 (0.022)	0.916 (0.012)	0.267 (0.007)
	BF	0.098 (0.013)	0.204 (0.018)	0.516 (0.022)	0.142 (0.016)	0.454 (0.022)	0.914 (0.013)	0.263 (0.007)
	AIC	0.092 (0.013)	0.166 (0.017)	0.520 (0.022)	0.122 (0.015)	0.396 (0.022)	0.918 (0.012)	0.249 (0.006)
	BIC	0.082 (0.012)	0.162 (0.016)	0.518 (0.022)	0.112 (0.014)	0.394 (0.022)	0.916 (0.012)	0.246 (0.006)
Gamma	BMA	0.094 (0.013)	0.246 (0.019)	0.550 (0.022)	0.150 (0.016)	0.518 (0.022)	0.956 (0.009)	0.284 (0.007)
	BF	0.090 (0.013)	0.230 (0.019)	0.550 (0.022)	0.148 (0.016)	0.496 (0.022)	0.958 (0.009)	0.279 (0.007)
	AIC	0.076 (0.012)	0.184 (0.017)	0.558 (0.022)	0.122 (0.015)	0.442 (0.022)	0.956 (0.009)	0.263 (0.007)
	BIC	0.068 (0.011)	0.180 (0.017)	0.556 (0.022)	0.114 (0.014)	0.430 (0.022)	0.954 (0.009)	0.259 (0.007)
Average	BMA	0.094 (0.013)	0.246 (0.019)	0.550 (0.022)	0.150 (0.016)	0.518 (0.022)	0.956 (0.009)	0.264 (0.003)
	BF	0.094 (0.013)	0.246 (0.019)	0.550 (0.022)	0.150 (0.016)	0.518 (0.022)	0.956 (0.009)	0.260 (0.003)
	AIC	0.081 (0.005)	0.168 (0.007)	0.483 (0.010)	0.130 (0.007)	0.388 (0.010)	0.916 (0.006)	0.244 (0.003)
	BIC	0.073 (0.005)	0.164 (0.007)	0.482 (0.010)	0.119 (0.006)	0.379 (0.010)	0.915 (0.006)	0.240 (0.003)

Table 23: Confidence intervals coverage (SE) for each simulation condition (Part 1) comparing Bayesian model-averaging (BMA) to model selection using information (AIC, BIC), and model selection using Bayes factors (BF), including the parametric models corresponding to the true data generating process. Rows correspond to different simulation conditions according to the true parametric family (Exp = exponential, Weib = Weibull, Log-N = log-normal, Log-L = Log-logistic, Gamma = gamma). Average corresponds to the results aggregated across distributions (last row), log(AF) × N (last column), or the whole simulation (bottom right corner).

**% CI**		log(AF) = -0.20			log(AF) = 0		
		N = 50	N = 200	N = 1000	N = 50	N = 200	N = 1000
Exp	BMA	0.992 (0.004)	0.978 (0.007)	0.936 (0.011)	0.982 (0.006)	0.948 (0.010)	0.936 (0.011)
	BF	0.986 (0.005)	0.976 (0.007)	0.934 (0.011)	0.982 (0.006)	0.956 (0.009)	0.934 (0.011)
	AIC	0.966 (0.008)	0.964 (0.008)	0.932 (0.011)	0.952 (0.010)	0.946 (0.010)	0.932 (0.011)
	BIC	0.978 (0.007)	0.966 (0.008)	0.938 (0.011)	0.962 (0.009)	0.948 (0.010)	0.932 (0.011)
Weib	BMA	0.980 (0.006)	0.956 (0.009)	0.954 (0.009)	0.984 (0.006)	0.962 (0.009)	0.944 (0.010)
	BF	0.986 (0.005)	0.960 (0.009)	0.954 (0.009)	0.986 (0.005)	0.962 (0.009)	0.942 (0.010)
	AIC	0.964 (0.008)	0.946 (0.010)	0.952 (0.010)	0.940 (0.011)	0.954 (0.009)	0.938 (0.011)
	BIC	0.970 (0.008)	0.952 (0.010)	0.954 (0.009)	0.956 (0.009)	0.958 (0.009)	0.940 (0.011)
Log-N	BMA	0.974 (0.007)	0.958 (0.009)	0.962 (0.009)	0.986 (0.005)	0.954 (0.009)	0.956 (0.009)
	BF	0.974 (0.007)	0.962 (0.009)	0.960 (0.009)	0.988 (0.005)	0.960 (0.009)	0.956 (0.009)
	AIC	0.958 (0.009)	0.948 (0.010)	0.954 (0.009)	0.968 (0.008)	0.942 (0.010)	0.958 (0.009)
	BIC	0.962 (0.009)	0.942 (0.010)	0.954 (0.009)	0.976 (0.007)	0.936 (0.011)	0.958 (0.009)
Log-L	BMA	0.970 (0.008)	0.954 (0.009)	0.946 (0.010)	0.978 (0.007)	0.954 (0.009)	0.958 (0.009)
	BF	0.968 (0.008)	0.956 (0.009)	0.948 (0.010)	0.976 (0.007)	0.952 (0.010)	0.954 (0.009)
	AIC	0.950 (0.010)	0.942 (0.010)	0.942 (0.010)	0.950 (0.010)	0.950 (0.010)	0.956 (0.009)
	BIC	0.954 (0.009)	0.944 (0.010)	0.942 (0.010)	0.956 (0.009)	0.952 (0.010)	0.956 (0.009)
Gamma	BMA	0.984 (0.006)	0.950 (0.010)	0.952 (0.010)	0.984 (0.006)	0.958 (0.009)	0.942 (0.010)
	BF	0.982 (0.006)	0.950 (0.010)	0.952 (0.010)	0.982 (0.006)	0.956 (0.009)	0.940 (0.011)
	AIC	0.968 (0.008)	0.934 (0.011)	0.950 (0.010)	0.962 (0.009)	0.954 (0.009)	0.942 (0.010)
	BIC	0.972 (0.007)	0.940 (0.011)	0.948 (0.010)	0.968 (0.008)	0.952 (0.010)	0.942 (0.010)
Average	BMA	0.980 (0.003)	0.959 (0.004)	0.950 (0.004)	0.983 (0.003)	0.955 (0.004)	0.947 (0.004)
	BF	0.979 (0.003)	0.961 (0.004)	0.950 (0.004)	0.983 (0.003)	0.957 (0.004)	0.945 (0.005)
	AIC	0.961 (0.004)	0.947 (0.004)	0.946 (0.005)	0.954 (0.004)	0.949 (0.004)	0.945 (0.005)
	BIC	0.967 (0.004)	0.949 (0.004)	0.947 (0.004)	0.964 (0.004)	0.949 (0.004)	0.946 (0.005)

Table 24: Confidence intervals coverage (SE) for each simulation condition (Part 2) comparing Bayesian model-averaging (BMA) to model selection using information (AIC, BIC), and model selection using Bayes factors (BF), including the parametric models corresponding to the true data generating process. Rows correspond to different simulation conditions according to the true parametric family (Exp = exponential, Weib = Weibull, Log-N = log-normal, Log-L = Log-logistic, Gamma = gamma). Average corresponds to the results aggregated across distributions (last row), log(AF) × N (last column), or the whole simulation (bottom right corner).

**% CI**		log(AF) = 0.20			log(AF) = 0.40			Average
		N = 50	N = 200	N = 1000	N = 50	N = 200	N = 1000	
Exp	BMA	0.980 (0.006)	0.952 (0.010)	0.960 (0.009)	0.984 (0.006)	0.958 (0.009)	0.952 (0.010)	0.963 (0.002)
	BF	0.982 (0.006)	0.954 (0.009)	0.960 (0.009)	0.980 (0.006)	0.956 (0.009)	0.952 (0.010)	0.963 (0.002)
	AIC	0.972 (0.007)	0.946 (0.010)	0.956 (0.009)	0.970 (0.008)	0.946 (0.010)	0.948 (0.010)	0.953 (0.003)
	BIC	0.972 (0.007)	0.950 (0.010)	0.960 (0.009)	0.970 (0.008)	0.956 (0.009)	0.956 (0.009)	0.957 (0.003)
Weib	BMA	0.988 (0.005)	0.972 (0.007)	0.944 (0.010)	0.980 (0.006)	0.964 (0.008)	0.964 (0.008)	0.966 (0.002)
	BF	0.984 (0.006)	0.972 (0.007)	0.942 (0.010)	0.986 (0.005)	0.964 (0.008)	0.966 (0.008)	0.967 (0.002)
	AIC	0.974 (0.007)	0.956 (0.009)	0.940 (0.011)	0.978 (0.007)	0.962 (0.009)	0.964 (0.008)	0.956 (0.003)
	BIC	0.974 (0.007)	0.962 (0.009)	0.940 (0.011)	0.978 (0.007)	0.956 (0.009)	0.960 (0.009)	0.958 (0.003)
Log-N	BMA	0.986 (0.005)	0.970 (0.008)	0.978 (0.007)	0.980 (0.006)	0.966 (0.008)	0.938 (0.011)	0.967 (0.002)
	BF	0.986 (0.005)	0.980 (0.006)	0.978 (0.007)	0.980 (0.006)	0.962 (0.009)	0.942 (0.010)	0.969 (0.002)
	AIC	0.964 (0.008)	0.964 (0.008)	0.976 (0.007)	0.954 (0.009)	0.952 (0.010)	0.936 (0.011)	0.956 (0.003)
	BIC	0.972 (0.007)	0.958 (0.009)	0.976 (0.007)	0.964 (0.008)	0.952 (0.010)	0.936 (0.011)	0.957 (0.003)
Log-L	BMA	0.976 (0.007)	0.966 (0.008)	0.946 (0.010)	0.984 (0.006)	0.958 (0.009)	0.952 (0.010)	0.962 (0.002)
	BF	0.980 (0.006)	0.972 (0.007)	0.944 (0.010)	0.978 (0.007)	0.954 (0.009)	0.952 (0.010)	0.961 (0.002)
	AIC	0.964 (0.008)	0.952 (0.010)	0.940 (0.011)	0.962 (0.009)	0.944 (0.010)	0.948 (0.010)	0.950 (0.003)
	BIC	0.966 (0.008)	0.956 (0.009)	0.940 (0.011)	0.964 (0.008)	0.944 (0.010)	0.950 (0.010)	0.952 (0.003)
Gamma	BMA	0.982 (0.006)	0.954 (0.009)	0.944 (0.010)	0.980 (0.006)	0.970 (0.008)	0.966 (0.008)	0.964 (0.002)
	BF	0.984 (0.006)	0.952 (0.010)	0.940 (0.011)	0.982 (0.006)	0.966 (0.008)	0.964 (0.008)	0.963 (0.002)
	AIC	0.958 (0.009)	0.940 (0.011)	0.952 (0.010)	0.964 (0.008)	0.956 (0.009)	0.962 (0.009)	0.954 (0.003)
	BIC	0.970 (0.008)	0.948 (0.010)	0.944 (0.010)	0.968 (0.008)	0.958 (0.009)	0.956 (0.009)	0.956 (0.003)
Average	BMA	0.982 (0.003)	0.963 (0.004)	0.954 (0.004)	0.982 (0.003)	0.963 (0.004)	0.954 (0.004)	0.964 (0.001)
	BF	0.983 (0.003)	0.966 (0.004)	0.953 (0.004)	0.981 (0.003)	0.960 (0.004)	0.955 (0.004)	0.964 (0.001)
	AIC	0.966 (0.004)	0.952 (0.004)	0.953 (0.004)	0.966 (0.004)	0.952 (0.004)	0.952 (0.004)	0.954 (0.001)
	BIC	0.971 (0.003)	0.955 (0.004)	0.952 (0.004)	0.969 (0.003)	0.953 (0.004)	0.952 (0.004)	0.956 (0.001)

Table 25: Bias (SE) of predicted mean survival in 20 years for each simulation condition (Part 1) comparing Bayesian model-averaging (BMA) to model selection using information (AIC, BIC), and model selection using Bayes factors (BF), including the parametric models corresponding to the true data generating process. Rows correspond to different simulation conditions according to the true parametric family (Exp = exponential, Weib = Weibull, Log-N = log-normal, Log-L = Log-logistic, Gamma = gamma). Average corresponds to the results aggregated across distributions (last row), log(AF) × N (last column), or the whole simulation (bottom right corner).

**Bias**		log(AF) = -0.20			log(AF) = 0		
		N = 50	N = 200	N = 1000	N = 50	N = 200	N = 1000
Exp	BMA	0.139 (0.003)	0.074 (0.002)	0.027 (0.001)	0.160 (0.003)	0.087 (0.002)	0.032 (0.001)
	BF	0.161 (0.005)	0.061 (0.003)	0.015 (0.002)	0.187 (0.005)	0.077 (0.003)	0.022 (0.002)
	AIC	0.065 (0.005)	0.034 (0.003)	0.021 (0.002)	0.085 (0.005)	0.046 (0.003)	0.027 (0.002)
	BIC	0.045 (0.004)	0.009 (0.002)	0.002 (0.001)	0.062 (0.005)	0.016 (0.002)	0.006 (0.001)
Weib	BMA	0.173 (0.003)	0.099 (0.002)	0.052 (0.001)	0.187 (0.003)	0.119 (0.002)	0.065 (0.001)
	BF	0.194 (0.004)	0.103 (0.003)	0.050 (0.002)	0.212 (0.005)	0.124 (0.003)	0.067 (0.002)
	AIC	0.092 (0.004)	0.069 (0.003)	0.035 (0.002)	0.097 (0.004)	0.081 (0.003)	0.049 (0.002)
	BIC	0.090 (0.004)	0.064 (0.002)	0.041 (0.002)	0.100 (0.004)	0.079 (0.002)	0.057 (0.002)
Log-N	BMA	-0.004 (0.003)	-0.043 (0.002)	-0.011 (0.001)	0.004 (0.003)	-0.044 (0.002)	-0.017 (0.001)
	BF	0.044 (0.004)	0.005 (0.003)	0.003 (0.001)	0.057 (0.004)	0.010 (0.003)	0.001 (0.001)
	AIC	-0.085 (0.004)	-0.043 (0.003)	-0.008 (0.001)	-0.086 (0.004)	-0.047 (0.003)	-0.012 (0.001)
	BIC	-0.088 (0.004)	-0.088 (0.002)	-0.009 (0.001)	-0.091 (0.004)	-0.090 (0.002)	-0.014 (0.001)
Log-L	BMA	0.052 (0.003)	-0.010 (0.002)	-0.051 (0.001)	0.060 (0.003)	-0.004 (0.002)	-0.050 (0.002)
	BF	0.084 (0.004)	0.007 (0.003)	-0.031 (0.002)	0.093 (0.005)	0.016 (0.003)	-0.027 (0.002)
	AIC	-0.031 (0.004)	-0.031 (0.003)	-0.042 (0.002)	-0.032 (0.005)	-0.031 (0.003)	-0.042 (0.002)
	BIC	-0.028 (0.004)	-0.047 (0.002)	-0.048 (0.002)	-0.030 (0.005)	-0.045 (0.002)	-0.048 (0.002)
Gamma	BMA	0.167 (0.003)	0.092 (0.002)	0.037 (0.001)	0.184 (0.003)	0.106 (0.002)	0.053 (0.001)
	BF	0.189 (0.004)	0.098 (0.003)	0.038 (0.002)	0.209 (0.005)	0.120 (0.003)	0.056 (0.002)
	AIC	0.085 (0.004)	0.064 (0.003)	0.027 (0.002)	0.092 (0.005)	0.071 (0.003)	0.040 (0.002)
	BIC	0.084 (0.004)	0.059 (0.002)	0.028 (0.002)	0.095 (0.005)	0.065 (0.002)	0.043 (0.002)
Average	BMA	0.106 (0.002)	0.043 (0.001)	0.011 (0.001)	0.119 (0.002)	0.053 (0.001)	0.017 (0.001)
	BF	0.134 (0.002)	0.055 (0.001)	0.015 (0.001)	0.152 (0.002)	0.069 (0.002)	0.024 (0.001)
	AIC	0.025 (0.002)	0.019 (0.001)	0.007 (0.001)	0.031 (0.002)	0.024 (0.002)	0.012 (0.001)
	BIC	0.021 (0.002)	0.000 (0.001)	0.003 (0.001)	0.027 (0.002)	0.005 (0.001)	0.009 (0.001)

Table 26: Bias (SE) of predicted mean survival in 20 years for each simulation condition (Part 2) comparing Bayesian model-averaging (BMA) to model selection using information (AIC, BIC), and model selection using Bayes factors (BF), including the parametric models corresponding to the true data generating process. Rows correspond to different simulation conditions according to the true parametric family (Exp = exponential, Weib = Weibull, Log-N = log-normal, Log-L = Log-logistic, Gamma = gamma). Average corresponds to the results aggregated across distributions (last row), log(AF) × N (last column), or the whole simulation (bottom right corner).

**Bias**		log(AF) = 0.20			log(AF) = 0.40			Average
		N = 50	N = 200	N = 1000	N = 50	N = 200	N = 1000	
Exp	BMA	0.143 (0.004)	0.084 (0.003)	0.033 (0.002)	0.145 (0.004)	0.080 (0.003)	0.036 (0.002)	0.087 (0.001)
	BF	0.174 (0.005)	0.077 (0.004)	0.022 (0.002)	0.175 (0.005)	0.072 (0.004)	0.021 (0.002)	0.089 (0.001)
	AIC	0.067 (0.006)	0.048 (0.004)	0.025 (0.002)	0.073 (0.006)	0.040 (0.004)	0.028 (0.002)	0.047 (0.001)
	BIC	0.051 (0.006)	0.014 (0.002)	0.005 (0.001)	0.056 (0.006)	0.008 (0.003)	0.006 (0.001)	0.023 (0.001)
Weib	BMA	0.198 (0.003)	0.130 (0.002)	0.071 (0.001)	0.212 (0.004)	0.139 (0.003)	0.075 (0.002)	0.127 (0.001)
	BF	0.221 (0.005)	0.139 (0.003)	0.069 (0.002)	0.239 (0.005)	0.146 (0.003)	0.067 (0.002)	0.136 (0.001)
	AIC	0.100 (0.005)	0.092 (0.003)	0.048 (0.002)	0.117 (0.006)	0.095 (0.004)	0.048 (0.003)	0.077 (0.001)
	BIC	0.106 (0.005)	0.087 (0.002)	0.061 (0.002)	0.127 (0.006)	0.099 (0.003)	0.063 (0.002)	0.081 (0.001)
Log-N	BMA	0.003 (0.004)	-0.047 (0.002)	-0.018 (0.002)	0.013 (0.004)	-0.047 (0.002)	-0.026 (0.002)	-0.020 (0.001)
	BF	0.057 (0.004)	0.014 (0.003)	0.005 (0.001)	0.067 (0.004)	0.008 (0.003)	-0.002 (0.002)	0.023 (0.001)
	AIC	-0.089 (0.005)	-0.053 (0.003)	-0.011 (0.002)	-0.085 (0.006)	-0.056 (0.003)	-0.019 (0.002)	-0.049 (0.001)
	BIC	-0.085 (0.005)	-0.090 (0.002)	-0.012 (0.002)	-0.070 (0.006)	-0.092 (0.003)	-0.020 (0.002)	-0.062 (0.001)
Log-L	BMA	0.072 (0.004)	0.006 (0.002)	-0.045 (0.002)	0.088 (0.004)	0.008 (0.002)	-0.035 (0.002)	0.008 (0.001)
	BF	0.109 (0.005)	0.032 (0.003)	-0.025 (0.003)	0.127 (0.004)	0.031 (0.003)	-0.017 (0.003)	0.033 (0.001)
	AIC	-0.024 (0.005)	-0.020 (0.003)	-0.047 (0.003)	-0.007 (0.006)	-0.037 (0.004)	-0.041 (0.003)	-0.032 (0.001)
	BIC	-0.017 (0.005)	-0.040 (0.002)	-0.047 (0.003)	0.008 (0.006)	-0.034 (0.003)	-0.039 (0.003)	-0.035 (0.001)
Gamma	BMA	0.196 (0.004)	0.116 (0.002)	0.054 (0.001)	0.196 (0.004)	0.123 (0.002)	0.064 (0.002)	0.116 (0.001)
	BF	0.223 (0.005)	0.136 (0.003)	0.058 (0.002)	0.223 (0.005)	0.138 (0.003)	0.069 (0.003)	0.130 (0.001)
	AIC	0.103 (0.005)	0.077 (0.003)	0.035 (0.002)	0.108 (0.006)	0.080 (0.003)	0.044 (0.003)	0.069 (0.001)
	BIC	0.110 (0.005)	0.077 (0.002)	0.043 (0.002)	0.117 (0.006)	0.085 (0.003)	0.051 (0.003)	0.072 (0.001)
Average	BMA	0.122 (0.002)	0.058 (0.001)	0.019 (0.001)	0.131 (0.002)	0.061 (0.002)	0.023 (0.001)	0.063 (0.000)
	BF	0.157 (0.002)	0.080 (0.002)	0.026 (0.001)	0.166 (0.002)	0.079 (0.002)	0.028 (0.001)	0.082 (0.001)
	AIC	0.031 (0.003)	0.029 (0.002)	0.010 (0.001)	0.041 (0.003)	0.024 (0.002)	0.012 (0.001)	0.022 (0.001)
	BIC	0.033 (0.003)	0.010 (0.001)	0.010 (0.001)	0.048 (0.003)	0.013 (0.002)	0.012 (0.001)	0.016 (0.001)

Table 27: RMSE (SE) of predicted mean survival in 20 years for each simulation condition (Part 1) comparing Bayesian model-averaging (BMA) to model selection using information (AIC, BIC), and model selection using Bayes factors (BF), including the parametric models corresponding to the true data generating process. Rows correspond to different simulation conditions according to the true parametric family (Exp = exponential, Weib = Weibull, Log-N = log-normal, Log-L = Log-logistic, Gamma = gamma). Average corresponds to the results aggregated across distributions (last row), log(AF) × N (last column), or the whole simulation (bottom right corner).

**RMSE**		log(AF) = -0.20			log(AF) = 0		
		N = 50	N = 200	N = 1000	N = 50	N = 200	N = 1000
Exp	BMA	0.172 (0.003)	0.099 (0.002)	0.047 (0.002)	0.192 (0.003)	0.111 (0.002)	0.052 (0.002)
	BF	0.215 (0.004)	0.118 (0.004)	0.053 (0.003)	0.236 (0.004)	0.132 (0.003)	0.062 (0.003)
	AIC	0.169 (0.007)	0.101 (0.004)	0.064 (0.003)	0.193 (0.006)	0.112 (0.004)	0.073 (0.003)
	BIC	0.149 (0.007)	0.058 (0.002)	0.027 (0.002)	0.175 (0.007)	0.071 (0.003)	0.036 (0.002)
Weib	BMA	0.197 (0.003)	0.113 (0.002)	0.063 (0.001)	0.214 (0.003)	0.133 (0.002)	0.077 (0.002)
	BF	0.236 (0.004)	0.134 (0.003)	0.081 (0.002)	0.257 (0.004)	0.157 (0.003)	0.100 (0.002)
	AIC	0.153 (0.005)	0.109 (0.003)	0.070 (0.002)	0.169 (0.006)	0.125 (0.003)	0.088 (0.002)
	BIC	0.150 (0.006)	0.088 (0.002)	0.068 (0.002)	0.169 (0.006)	0.102 (0.002)	0.085 (0.002)
Log-N	BMA	0.099 (0.002)	0.073 (0.001)	0.044 (0.001)	0.103 (0.003)	0.075 (0.001)	0.047 (0.001)
	BF	0.144 (0.003)	0.090 (0.002)	0.040 (0.002)	0.147 (0.003)	0.090 (0.002)	0.042 (0.002)
	AIC	0.158 (0.004)	0.098 (0.002)	0.042 (0.002)	0.164 (0.003)	0.102 (0.002)	0.046 (0.002)
	BIC	0.164 (0.004)	0.111 (0.002)	0.043 (0.002)	0.167 (0.003)	0.113 (0.002)	0.047 (0.002)
Log-L	BMA	0.111 (0.003)	0.060 (0.001)	0.068 (0.001)	0.121 (0.004)	0.060 (0.001)	0.069 (0.001)
	BF	0.158 (0.003)	0.093 (0.002)	0.078 (0.001)	0.173 (0.004)	0.102 (0.002)	0.082 (0.001)
	AIC	0.138 (0.005)	0.095 (0.002)	0.083 (0.001)	0.146 (0.005)	0.101 (0.002)	0.089 (0.002)
	BIC	0.138 (0.005)	0.079 (0.001)	0.081 (0.001)	0.147 (0.006)	0.082 (0.002)	0.086 (0.002)
Gamma	BMA	0.193 (0.003)	0.107 (0.002)	0.052 (0.001)	0.211 (0.004)	0.122 (0.002)	0.070 (0.002)
	BF	0.232 (0.004)	0.130 (0.003)	0.075 (0.002)	0.255 (0.004)	0.157 (0.003)	0.096 (0.002)
	AIC	0.157 (0.006)	0.105 (0.003)	0.069 (0.002)	0.172 (0.006)	0.121 (0.003)	0.087 (0.002)
	BIC	0.148 (0.006)	0.085 (0.002)	0.065 (0.002)	0.173 (0.006)	0.095 (0.002)	0.084 (0.002)
Average	BMA	0.160 (0.002)	0.093 (0.001)	0.055 (0.001)	0.175 (0.002)	0.104 (0.001)	0.064 (0.001)
	BF	0.201 (0.002)	0.114 (0.001)	0.067 (0.001)	0.218 (0.002)	0.131 (0.001)	0.079 (0.001)
	AIC	0.155 (0.003)	0.102 (0.001)	0.067 (0.001)	0.170 (0.003)	0.113 (0.001)	0.078 (0.001)
	BIC	0.150 (0.002)	0.086 (0.001)	0.060 (0.001)	0.166 (0.003)	0.094 (0.001)	0.071 (0.001)

Table 28: RMSE (SE) of predicted mean survival in 20 years for each simulation condition (Part 2) comparing Bayesian model-averaging (BMA) to model selection using information (AIC, BIC), and model selection using Bayes factors (BF), including the parametric models corresponding to the true data generating process. Rows correspond to different simulation conditions according to the true parametric family (Exp = exponential, Weib = Weibull, Log-N = log-normal, Log-L = Log-logistic, Gamma = gamma). Average corresponds to the results aggregated across distributions (last row), log(AF) × N (last column), or the whole simulation (bottom right corner).

**RMSE**		log(AF) = 0.20			log(AF) = 0.40			Average
		N = 50	N = 200	N = 1000	N = 50	N = 200	N = 1000	
Exp	BMA	0.182 (0.004)	0.116 (0.003)	0.061 (0.002)	0.190 (0.004)	0.121 (0.003)	0.068 (0.002)	0.129 (0.001)
	BF	0.228 (0.004)	0.139 (0.004)	0.069 (0.003)	0.229 (0.004)	0.140 (0.003)	0.066 (0.003)	0.156 (0.001)
	AIC	0.201 (0.007)	0.128 (0.004)	0.078 (0.003)	0.210 (0.007)	0.128 (0.004)	0.078 (0.003)	0.137 (0.002)
	BIC	0.183 (0.007)	0.079 (0.003)	0.041 (0.003)	0.197 (0.007)	0.085 (0.003)	0.046 (0.002)	0.113 (0.002)
Weib	BMA	0.224 (0.004)	0.147 (0.002)	0.085 (0.002)	0.243 (0.004)	0.161 (0.003)	0.092 (0.002)	0.157 (0.001)
	BF	0.263 (0.004)	0.171 (0.003)	0.103 (0.003)	0.281 (0.004)	0.179 (0.003)	0.104 (0.002)	0.185 (0.001)
	AIC	0.183 (0.007)	0.138 (0.003)	0.091 (0.002)	0.212 (0.007)	0.146 (0.004)	0.096 (0.002)	0.138 (0.002)
	BIC	0.185 (0.007)	0.113 (0.002)	0.093 (0.002)	0.219 (0.007)	0.135 (0.003)	0.098 (0.002)	0.133 (0.002)
Log-N	BMA	0.111 (0.003)	0.080 (0.001)	0.055 (0.002)	0.119 (0.003)	0.089 (0.002)	0.061 (0.002)	0.083 (0.001)
	BF	0.151 (0.003)	0.092 (0.002)	0.047 (0.002)	0.157 (0.003)	0.099 (0.002)	0.051 (0.002)	0.105 (0.001)
	AIC	0.183 (0.004)	0.112 (0.003)	0.054 (0.002)	0.194 (0.004)	0.120 (0.003)	0.059 (0.002)	0.123 (0.001)
	BIC	0.185 (0.004)	0.115 (0.002)	0.054 (0.002)	0.196 (0.004)	0.124 (0.002)	0.059 (0.002)	0.126 (0.001)
Log-L	BMA	0.133 (0.004)	0.070 (0.002)	0.072 (0.001)	0.145 (0.003)	0.078 (0.002)	0.071 (0.001)	0.093 (0.001)
	BF	0.182 (0.004)	0.109 (0.002)	0.090 (0.002)	0.190 (0.003)	0.112 (0.002)	0.090 (0.002)	0.128 (0.001)
	AIC	0.156 (0.005)	0.106 (0.002)	0.100 (0.002)	0.183 (0.006)	0.118 (0.002)	0.101 (0.002)	0.122 (0.001)
	BIC	0.158 (0.005)	0.084 (0.002)	0.093 (0.002)	0.185 (0.006)	0.100 (0.002)	0.092 (0.002)	0.116 (0.001)
Gamma	BMA	0.225 (0.004)	0.133 (0.002)	0.071 (0.002)	0.231 (0.004)	0.145 (0.002)	0.085 (0.002)	0.150 (0.001)
	BF	0.268 (0.004)	0.171 (0.003)	0.097 (0.002)	0.270 (0.004)	0.172 (0.003)	0.108 (0.002)	0.182 (0.001)
	AIC	0.198 (0.008)	0.126 (0.003)	0.085 (0.002)	0.217 (0.008)	0.136 (0.003)	0.097 (0.002)	0.138 (0.002)
	BIC	0.204 (0.008)	0.108 (0.002)	0.085 (0.002)	0.222 (0.007)	0.127 (0.003)	0.096 (0.002)	0.134 (0.002)
Average	BMA	0.181 (0.002)	0.113 (0.001)	0.070 (0.001)	0.192 (0.002)	0.123 (0.001)	0.076 (0.001)	0.126 (0.000)
	BF	0.223 (0.002)	0.140 (0.001)	0.084 (0.001)	0.230 (0.002)	0.144 (0.001)	0.087 (0.001)	0.154 (0.001)
	AIC	0.185 (0.003)	0.123 (0.001)	0.083 (0.001)	0.204 (0.003)	0.130 (0.001)	0.088 (0.001)	0.132 (0.001)
	BIC	0.184 (0.003)	0.101 (0.001)	0.076 (0.001)	0.204 (0.003)	0.116 (0.001)	0.081 (0.001)	0.125 (0.001)

Table 29: Probability (SE) of finding support for the null hypothesis (H_0), alternative hypothesis (H_1), or not reaching a decision (undecided) in the simulated sequential analysis for each simulation condition comparing Bayesian model-averaging (BMA) to model selection using information (AIC, BIC), model selection using Bayes factors (BF), including the parametric models corresponding to the true data generating process, Bayesian or frequentist survival model with the true data generating survival function (Oracle), and Cox proportional hazard model. Different rows under AIC / BIC / Cox proportional hazard model correspond to the number of steps in the sequential analysis with binding asymmetric boundaries, Hwang-Shih-DeCani spending function, and $\alpha = 0.05$ for one-sided test.

		log(AF) = -.20			log(AF) = 0		
		H_0	Undecided	H_1	H_0	Undecided	H_1
BMA		0.998 (0.002)	0.000 (0.000)	0.002 (0.002)	0.898 (0.014)	0.046 (0.009)	0.056 (0.010)
BF		0.998 (0.002)	0.000 (0.000)	0.002 (0.002)	0.890 (0.014)	0.050 (0.010)	0.060 (0.011)
	2	1.000 (0.000)	0.000 (0.000)	0.000 (0.000)	0.594 (0.022)	0.388 (0.022)	0.018 (0.006)
	4	1.000 (0.000)	0.000 (0.000)	0.000 (0.000)	0.858 (0.016)	0.118 (0.014)	0.024 (0.007)
AIC	5	1.000 (0.000)	0.000 (0.000)	0.000 (0.000)	0.898 (0.014)	0.082 (0.012)	0.020 (0.006)
	10	1.000 (0.000)	0.000 (0.000)	0.000 (0.000)	0.924 (0.012)	0.044 (0.009)	0.032 (0.008)
	20	1.000 (0.000)	0.000 (0.000)	0.000 (0.000)	0.932 (0.011)	0.040 (0.009)	0.028 (0.007)
	2	1.000 (0.000)	0.000 (0.000)	0.000 (0.000)	0.590 (0.022)	0.392 (0.022)	0.018 (0.006)
	4	1.000 (0.000)	0.000 (0.000)	0.000 (0.000)	0.854 (0.016)	0.120 (0.015)	0.026 (0.007)
BIC	5	1.000 (0.000)	0.000 (0.000)	0.000 (0.000)	0.898 (0.014)	0.082 (0.012)	0.020 (0.006)
	10	1.000 (0.000)	0.000 (0.000)	0.000 (0.000)	0.926 (0.012)	0.042 (0.009)	0.032 (0.008)
	20	1.000 (0.000)	0.000 (0.000)	0.000 (0.000)	0.932 (0.011)	0.040 (0.009)	0.028 (0.007)

		log(AF) = 0.20			log(AF) = 0.40		
		H_0	Undecided	H_1	H_0	Undecided	H_1
BMA		0.184 (0.017)	0.134 (0.015)	0.682 (0.021)	0.016 (0.006)	0.008 (0.004)	0.976 (0.007)
BF		0.164 (0.017)	0.152 (0.016)	0.684 (0.021)	0.016 (0.006)	0.008 (0.004)	0.976 (0.007)
	2	0.038 (0.009)	0.704 (0.020)	0.258 (0.020)	0.002 (0.002)	0.090 (0.013)	0.908 (0.013)
	4	0.082 (0.012)	0.336 (0.021)	0.582 (0.022)	0.004 (0.003)	0.020 (0.006)	0.976 (0.007)
AIC	5	0.100 (0.013)	0.266 (0.020)	0.634 (0.022)	0.002 (0.002)	0.022 (0.007)	0.976 (0.007)
	10	0.112 (0.014)	0.234 (0.019)	0.654 (0.021)	0.004 (0.003)	0.012 (0.005)	0.984 (0.006)
	20	0.112 (0.014)	0.208 (0.018)	0.680 (0.021)	0.006 (0.003)	0.012 (0.005)	0.982 (0.006)
	2	0.038 (0.009)	0.704 (0.020)	0.258 (0.020)	0.002 (0.002)	0.086 (0.013)	0.912 (0.013)
	4	0.082 (0.012)	0.334 (0.021)	0.584 (0.022)	0.004 (0.003)	0.020 (0.006)	0.976 (0.007)
BIC	5	0.102 (0.014)	0.264 (0.020)	0.634 (0.022)	0.002 (0.002)	0.022 (0.007)	0.976 (0.007)
	10	0.114 (0.014)	0.232 (0.019)	0.654 (0.021)	0.004 (0.003)	0.012 (0.005)	0.984 (0.006)
	20	0.116 (0.014)	0.204 (0.018)	0.680 (0.021)	0.006 (0.003)	0.012 (0.005)	0.982 (0.006)

Table 30: Mean time in months (SE) of finding support for either the null or alternative hypothesis for each simulation condition comparing Bayesian model-averaging (BMA) to model selection using information (AIC, BIC), model selection using Bayes factors (BF), including the parametric models corresponding to the true data generating process, Bayesian or frequentist survival model with the true data generating survival function (Oracle), and Cox proportional hazard model. Different rows under AIC / BIC / Cox proportional hazard model correspond to the number of steps in the sequential analysis with binding asymmetric boundaries, Hwang-Shih-DeCani spending function, and $\alpha = 0.05$ for one-sided test.

**Time**		log(AF) = -0.20		log(AF) = 0		Average		
		H_0	H_1	H_0	H_0	H_0	H_1	H_0 or H_1
BMA		5.3 (0.14)	4.0 (–)	10.4 (0.34)	7.0 (0.96)	7.7 (0.20)	6.9 (0.93)	7.7 (0.19)
BF		5.6 (0.15)	4.0 (–)	11.3 (0.40)	8.1 (0.97)	8.3 (0.22)	7.9 (0.95)	8.3 (0.22)
	2	16.9 (0.27)	– (–)	20.3 (0.55)	31.9 (7.04)	18.2 (0.27)	31.9 (7.04)	18.3 (0.28)
	4	10.8 (0.19)	– (–)	21.0 (0.46)	26.2 (5.16)	15.5 (0.29)	26.2 (5.16)	15.7 (0.29)
AIC	5	10.3 (0.20)	– (–)	20.9 (0.49)	34.4 (5.99)	15.3 (0.31)	34.4 (5.99)	15.5 (0.31)
	10	9.0 (0.18)	– (–)	20.1 (0.52)	35.9 (4.71)	14.3 (0.32)	35.9 (4.71)	14.7 (0.34)
	20	8.5 (0.18)	– (–)	19.7 (0.52)	28.9 (4.38)	13.9 (0.32)	28.9 (4.38)	14.1 (0.33)
	2	16.8 (0.25)	– (–)	20.4 (0.55)	31.9 (7.04)	18.1 (0.27)	31.9 (7.04)	18.3 (0.28)
	4	10.8 (0.19)	– (–)	20.9 (0.46)	25.0 (4.87)	15.4 (0.29)	25.0 (4.87)	15.6 (0.29)
BIC	5	10.2 (0.20)	– (–)	20.8 (0.48)	34.4 (5.99)	15.2 (0.30)	34.4 (5.99)	15.4 (0.31)
	10	9.0 (0.18)	– (–)	19.9 (0.51)	36.3 (4.65)	14.2 (0.32)	36.3 (4.65)	14.6 (0.33)
	20	8.4 (0.18)	– (–)	19.7 (0.52)	28.6 (4.35)	13.9 (0.32)	28.6 (4.35)	14.1 (0.33)

**Time**		log(AF) = 0.20		log(AF) = 0.40		Average		
		H_0	H_1	H_0	H_0	H_0	H_1	H_0 or H_1
BMA		11.0 (0.76)	14.0 (0.54)	7.8 (1.31)	8.7 (0.26)	10.7 (0.71)	10.8 (0.29)	10.8 (0.27)
BF		11.5 (0.88)	13.8 (0.53)	8.8 (1.31)	8.7 (0.26)	11.3 (0.81)	10.8 (0.28)	10.8 (0.26)
	2	20.3 (0.31)	20.4 (0.12)	24.4 (–)	22.7 (0.07)	20.5 (0.36)	22.2 (0.07)	22.1 (0.07)
	4	26.9 (1.81)	30.1 (0.67)	17.8 (6.66)	21.5 (0.49)	26.5 (1.76)	24.7 (0.42)	24.8 (0.41)
AIC	5	29.7 (2.19)	32.5 (0.83)	30.1 (–)	19.8 (0.40)	29.8 (2.15)	24.8 (0.46)	25.1 (0.46)
	10	28.3 (2.18)	29.3 (0.73)	14.5 (9.96)	17.8 (0.34)	27.9 (2.15)	22.4 (0.41)	22.7 (0.41)
	20	29.7 (2.15)	28.8 (0.71)	12.5 (5.07)	16.9 (0.32)	28.8 (2.11)	21.7 (0.40)	22.2 (0.41)
	2	20.3 (0.31)	20.4 (0.12)	24.4 (–)	22.7 (0.07)	20.5 (0.36)	22.2 (0.07)	22.1 (0.07)
	4	27.7 (1.93)	30.0 (0.67)	17.8 (6.66)	21.3 (0.48)	27.2 (1.88)	24.5 (0.42)	24.7 (0.41)
BIC	5	29.8 (2.20)	32.2 (0.84)	30.1 (–)	19.4 (0.41)	29.8 (2.16)	24.5 (0.47)	24.8 (0.46)
	10	28.0 (2.17)	29.3 (0.74)	14.5 (9.96)	17.6 (0.34)	27.5 (2.13)	22.2 (0.41)	22.6 (0.41)
	20	28.9 (2.08)	28.5 (0.72)	11.9 (5.57)	16.7 (0.33)	28.1 (2.05)	21.5 (0.41)	21.9 (0.41)
